# Molecular Evolution of Broadly Neutralizing Llama Antibodies to the CD4-Binding Site of HIV-1

**DOI:** 10.1371/journal.ppat.1004552

**Published:** 2014-12-18

**Authors:** Laura E. McCoy, Lucy Rutten, Dan Frampton, Ian Anderson, Luke Granger, Rachael Bashford-Rogers, Gillian Dekkers, Nika M. Strokappe, Michael S. Seaman, Willie Koh, Vanina Grippo, Alexander Kliche, Theo Verrips, Paul Kellam, Ariberto Fassati, Robin A. Weiss

**Affiliations:** 1 Wohl Virion Centre and Medical Research Council (MRC) Centre for Medical Molecular Virology, Division of Infection and Immunity, University College London, London, United Kingdom; 2 QVQ B.V., Utrecht, The Netherlands; 3 Department of Infectious Diseases, King's College London School of Medicine, Guy's Hospital, London, United Kingdom; 4 Wellcome Trust Sanger Institute, Cambridge, United Kingdom; 5 Center for Virology and Vaccine Research, Beth Israel Deaconess Medical Center, Harvard Medical School, Boston, Massachusetts, United States of America; 6 Centro de Virología Animal, Instituto de Ciencia y Tecnología Dr. César Milstein, Consejo Nacional de Investigaciones Científicas y Técnicas (CONICET), Buenos Aires, Argentina; 7 Institute of Medical Microbiology, University of Regensburg, Regensburg, Germany; University of Miami, United States of America

## Abstract

To date, no immunization of humans or animals has elicited broadly neutralizing sera able to prevent HIV-1 transmission; however, elicitation of broad and potent heavy chain only antibodies (HCAb) has previously been reported in llamas. In this study, the anti-HIV immune responses in immunized llamas were studied via deep sequencing analysis using broadly neutralizing monoclonal HCAbs as a guides. Distinct neutralizing antibody lineages were identified in each animal, including two defined by novel antibodies (as variable regions called VHH) identified by robotic screening of over 6000 clones. The combined application of five VHH against viruses from clades A, B, C and CRF_AG resulted in neutralization as potent as any of the VHH individually and a predicted 100% coverage with a median IC50 of 0.17 µg/ml for the panel of 60 viruses tested. Molecular analysis of the VHH repertoires of two sets of immunized animals showed that each neutralizing lineage was only observed following immunization, demonstrating that they were elicited *de novo*. Our results show that immunization can induce potent and broadly neutralizing antibodies in llamas with features similar to human antibodies and provide a framework to analyze the effectiveness of immunization protocols.

## Author Summary

Developing a vaccine against HIV-1 is a priority, but it remains unclear whether immunizations in humans can elicit potent broadly neutralizing antibodies able to prevent HIV-1 transmission. Llamas possess heavy chain only antibodies and conventional heavy and light chain antibodies. We previously reported the heavy chain only antibody J3, which potently neutralizes more than 95% of HIV strains, and was induced by immunization. Here we immunized two further llamas and elicited three novel broadly neutralizing heavy chain only antibodies, which were identified by high-throughput screening. These neutralizing llama antibodies target different areas of the CD4-binding site of the virus, therefore breadth and potency are increased when they are used in combination. To gain greater understanding of how the llama immunizations worked, deep sequencing of the HIV binding region of the antibodies was performed. This revealed that the antibodies were matured fully only in response to the protein immunogens. Furthermore, the VHH elicited in different animals, while sharing functional hallmarks, were encoded by distinct sequences and thus could not have been identified by a deep sequencing analysis alone. Our results show that immunization can potentially induce protective antibodies in llamas and provide a method to more extensively evaluate immunization studies.

## Introduction

HIV transmission remains a huge global public health problem (www.UNAIDS.org). To reduce spread of the virus new prevention methods are being developed based on recent advances in the molecular virology of HIV. In addition to the expanded use of ARVs in new modalities such as pre-exposure prophylaxis and microbicides [1], this includes commensal microbicides [2], gene therapies [3], [4] and vaccines [5], [6], [7]. Recent major advances in identifying broad and potent HIV neutralizing monoclonal antibodies (mAb) provide invaluable reagents for the development of these strategies to prevent HIV infection. It is well-established that passive infusion of neutralizing mAb can prevent SHIV infection [8], [9], [10], [11], [12], [13] and recently it was shown that a mAb can treat infection in non-human primate (NHP) models [14]. This combined with the success of antibody-inducing vaccines against other pathogens suggested that a vaccine that can induce neutralizing antibodies at sufficient titers could protect against HIV [15]. While viral escape has not been observed in the NHP models of immunoprophylaxis described above, virus can evolve in response to neutralizing antibodies in HIV-positive patients [16], [17] and early studies showed escape of patient virus from passive antibody-mediated protection when a single mAb was used [18]. However, recent *in vitro* work has shown increased neutralization coverage can be achieved by combining the newly identified broadly neutralizing mAb [19], [20]. These findings highlight the need to (a) induce multiple neutralizing antibody lineages for a protective antibody-based vaccine and (b) the potential need to use combinations of purified mAb in therapeutic or prophylactic settings.

To date, immunizations in human and animal models have yielded antibodies with only limited ability to neutralize HIV [21], [22], [23], [24], except llama heavy chain only antibodies (HCAbs) isolated as individual variable regions (VHH) [25]. Previously, we isolated the VHH J3 from a llama immunized with HIV envelope glycoprotein (Env) in the form of trimeric recombinant gp140 subunits and found it neutralized 96 of 100 HIV-1 strains by targeting the CD4-binding site (CD4bs) [26]. Llamas, along with other camelids have HCAb in additional to conventional heavy and light chain antibodies, [27]. The VHH is the total antigen binding domain or active site of these single-headed immunglobulins (IgG) and llama antibodies have been studied in this single domain form due to the dual advantages of bacterial/yeast expression and their high levels of thermal and pH stability [28]. As stable single domains, VHH have advantages for use in microbicides both in terms of gels [28] and protection by commensal bacteria [29].

In this study, we identified three new broadly neutralizing VHH, which bind to the CD4-binding site of the Env subunit gp120 and bind the molecular probe used to isolate VRC01. Deep sequencing of the VHH phage libraries generated from a set of llamas, which received two different immunization protocols, showed that the new VHH and the previously described anti-HIV VHH J3 [26] were induced by immunization (Table 1). As such the HIV llama vaccination model is robust and reproducible and demonstrates the potential of a mammalian immune system to produce broadly HIV neutralizing antibodies in response to immunization. We also demonstrate that multiple broadly neutralizing antibody lineages can be raised against HIV in the llama HCAb model and that, when combined as purified VHH, they provide enhanced breadth and potency of neutralization.

**Table 1:** Summary of llama immunizations.

**Immunogens**	96ZM965.01 and R2		92UG037 and CN54	
**Animal**	Llama 1	Llama 3	Llama 8	Llama 9
**VHH**	B9, A14	B21	J3	3E3

Each llama received the indicated gp140 immunogen as described in the materials and methods and [26].

## Results

### Identification of novel HIV neutralizing VHH from immunized llamas

Two llamas (*Lama glama*) (designated Llama 1 and Llama 3) were immunized via intramuscular injection of DNA encoding R2 and 96ZM651.02 gp160, and twice more with this DNA in combination with virus-like particles (VLP) bearing R2 and 96ZM651.02 gp145 protein (Table S1 in S1 Text). Sera taken one week after the last immunization (t = 54) showed measurable binding to homologous gp140 proteins in ELISA but no HIV neutralization activity in the TZM-bl assay (Figure S1 in S1 Text). Both llamas were subsequently immunized a further four times with soluble recombinant gp140 proteins again from R2 and 96ZM651.02 HIV strains. In contrast to non-neutralizing response at t = 54, post immune sera taken from both llamas one week after the final protein immunization (t = 174) neutralized HIV strains from clades A, B and C at 50% inhibitory dilutions (ID50) ranging from 1∶8 up to 1∶200 (Figure S1 in S1 Text). While the potency of these responses is modest, the breadth of activity included ‘difficult-to–neutralize’ strains (categorized as tier 2, where tier 1 is easiest and tier 3 is most difficult to neutralize) [30] from clades A and C, and titers of>1: 100 were seen for both llamas against the homologous strain 96ZM651.02. These serum neutralization titers^i^ were similar to those seen in our previous study where llama 8 was immunized with clade A and B/C gp140 proteins and produced the broadly neutralizing VHH J3 [26]. Thus the serum responses seen were considered strong enough to commence phage library construction and screening for individual VHH using the previously described direct neutralization protocol [26]. VHH clones were initially identified as neutralizing via 384 well robotic screening against two HIV pseudoviruses in the TZM-bl assay (see methods and materials). Initial hits were verified by repeated screening in the TZM-bl assay against a further three viruses. The robotic screening was designed to identify antibodies that neutralize tier 3 strains of HIV against which J3 is either partially or completely inactive by testing initial hits for the ability to neutralize these strains (Du172 and T257-31) at an early time point during the validation process.

**Figure 1:**
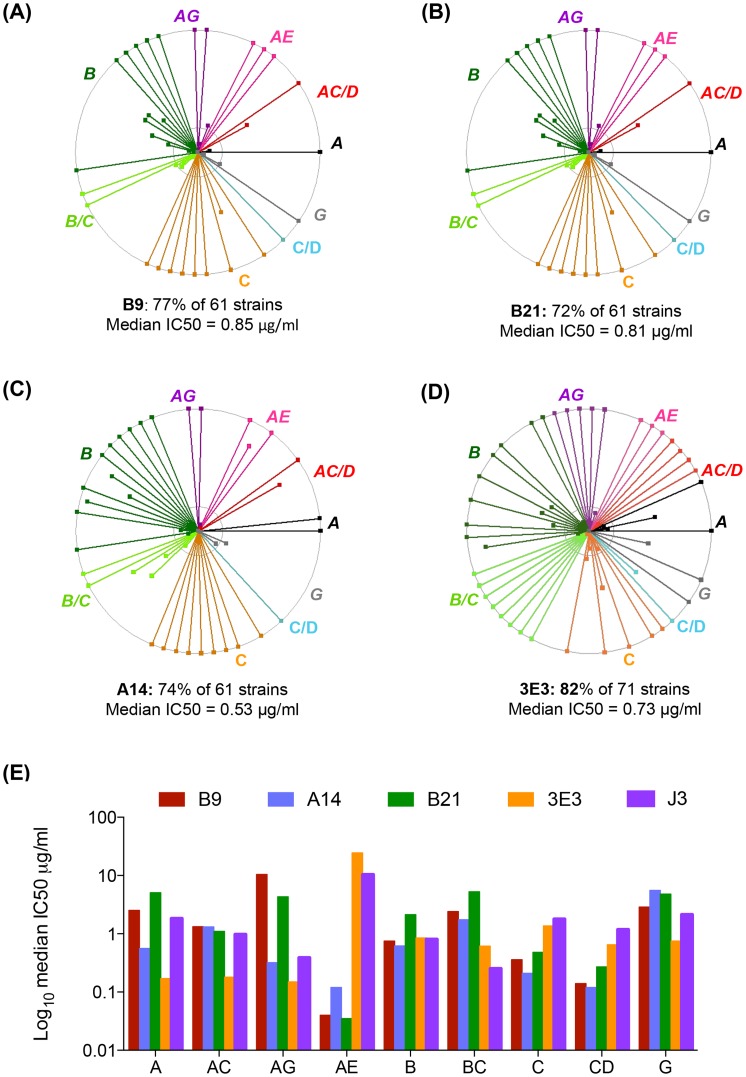
3E3, A14, B9 and B21 neutralization breadth and potency. (A, B, C, D) Each spoke represents a strain of HIV neutralized by B9 (A), B21 (B) A14 (C) and 3E3 (D) in the TZM-bl assay described in the Materials and Methods. Strains from different clades and CRF are color-coded according to the labels. The outer circle represents an IC50 of <1 µg/ml, the inner circle <5 µg/ml and the centre of the circle 50 µg/ml. legend. (E) Median IC50 µg/ml generated in the TZM-bl assay for each clade/CRF shown on the X-axis for the four VHH indicated in the color-coded legend. All viruses were assayed in duplicate to generate IC50 values.

Three VHH called A14, B9 and B21 were identified as neutralizing>1 tier 3 HIV pseudovirus and were subsequently purified and IC50 values calculated against sixty one viruses from a range of clades and circulating recombinant forms (CRF) (Table S2 in S1 Text). Overall, B9 was the broadest neutralizing VHH, blocking 77% of strains tested with a median 50% inhibitory concentration (IC50) of 0.85 µg/ml (Fig. 1A). A14 neutralized 74% of strains tested, but with slightly more potency with a median IC50 of 0.53 µg/ml (Fig. 1B). B21 neutralized 72% of strains with a median IC50 of 0.8 µg/ml (Fig. 1C). The ability of these VHH to neutralize virus was compared to the previously described VHH J3 and also to another newly identified VHH called 3E3, which was produced in the same immunization study as J3 but from a different llama (Table 1.). 3E3 was originally selected from the phagemid library derived from llama 9 by phage display via a competitive elution with soluble CD4 (sCD4) as described previously in [31]. However, re-selection of an identical clone by direct HIV-1 neutralization screening of the llama 9 phagemid library, as described in [26], indicated the breadth of this VHH and led to its further characterization. 3E3 neutralizes 82% of viral strains from a slightly larger panel of seventy one strains with a median IC50 of 0.73 µg/ml (Fig. 1D). However, the ability of 3E3 and the three new VHH to neutralize viruses within individual clades differs from that of J3, as reflected by the variation in median IC50 values for each clade (Fig. 1E). J3 and 3E3 have similar median IC50 values for each clade, with the exceptions of clade A and CRF AC where 3E3 has lower more potent IC50 values. There is more variation for the three VHH isolated following DNA/VLP/protein immunizations: for clade A and CRF CD, A14 has a log lower median IC50 than J3, similarly for clades C and CRF CD, A14 is more potent than J3, but for CRF BC J3 is more potent on average, while for clades G, B and CRF AG, all four VHH have similar median IC50 values. Notably for CRF_AE, A14, B9 and B21 have IC50 values more than 100-fold lower than J3.

### New VHH bind to the CD4bs differently to J3

**Figure 2:**
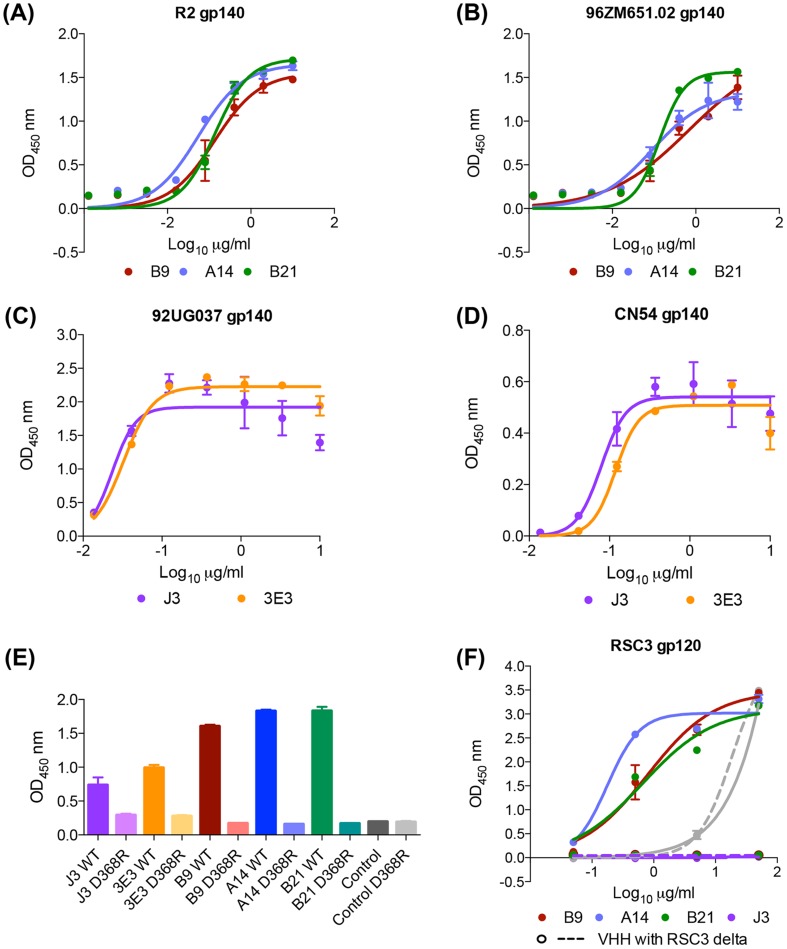
A14, B9, B21 binding to HIV ENV. VHH binding to immunogens (A) clade B gp140 R2, (B) clade C gp140 96ZM651.02, (C) clade A 92UG037 gp140, (D) CRF BC CN54 gp140, (E) YU2 WT and D368R gp120, and (F) RSC3 mutant and RSC3 delta mutant gp120, was assessed by ELISA as described in the Materials and Methods. The positive control for gp140 binding was J3 (McCoy et al. 2012) that for gp41 binding 2H10 [32]. The positive control for both RSC3 gp120 proteins was D47 a non-neutralizing RSC3-specific VHH (L McCoy unpublished data). All binding assays were carried out in duplicate and error bars represent standard deviation. These data are representative of at least three independent experiments.

B9, B21 and A14 all bind in a dose-dependent manner to the gp140 proteins used as the final immunogens (Fig. 2A,B). All three VHH were found to bind to gp140 derived from clade B HIV BX08 (gp140_BX08_), as does J3 but not to bind gp41 derived from clade B HIV IIIB (gp41_IIIB_) as does the VHH 2H10 [32](Figure S2A,B in S1 Text), thus the epitope(s) of these three VHH lie within gp120. 3E3 bound in a dose-dependent manner to the gp140 proteins (derived from HIV-1 strains 92UG037 and CN54) (Fig. 2C,B) but also did not bind gp41_IIIB_. To further pin point where the VHH binds, a gp120 protein derived from the YU2 strain of HIV with a CD4bs mutant unable to bind CD4 (D368R) was used. No binding was seen in ELISA to this mutant by 3E3, A14, B9 or B21 in comparison to the wild type YU2 gp120 (Fig. 2E). This confirmed that these VHH target the CD4bs of HIV Env as does J3 [26].

However, In contrast to J3, 3E3 and soluble CD4, A14, B9 and B21 all bind to an additional gp120 mutant called RSC3 [33] (Fig. 2F). The RSC3 recombinant gp120 protein was resurfaced to minimize recognition of non-CD4-binding site epitopes and used to probe supernatants of individual B cells from an elite neutralizer patient. This led to the isolation of VRC01, a mAb that neutralizes 90% of viruses tested [33]. However, neither A14, B9 nor B21 can bind to the RSCΔ mutant in which residue 371 has been altered to destroy the CD4 binding site (Fig. 2F) confirming their interaction with RSC3 is via the CD4-binding site. Crystallographic studies have revealed that VRC01, and related mAbs, bind to RSC3 by virtue of their angle of approach which is rotated 45° [34] relative to that of CD4 binding observed in a co-crystal with gp120 [35]. Structural studies are needed to assess whether these VHH bind in a similar fashion to VRC01. However, the total serum response is not dominated by these CD4-specific lineages as illustrated by the unaltered serum binding titres at all time points for both llamas against the RSC3 gp120 protein and the CD4-binding site mutant version RSC3delta (Figure S1C in S1 Text).

Analysis of the DNA and encoded amino acid sequences of A14 and B9 revealed that the first two are clonal variants which aligned most closely to the germ line denoted V_HH_ Vg (T Verrips, unpublished data) and that the sequence of B21 is highly divergent from both and also aligns to a different germ line V gene sequence Vu. B21, B9 and A14 belong to two distinct clonal families as compared with the previously described anti–HIV-1 VHH [26], [31], [36], [37] and 3E3 (Figure S3 in S1 Text). Furthermore, there is very limited similarity to the human germ line V gene VH1-2*02 precursor of the VRC01-like broadly neutralizing antibodies isolated from multiple patients. In contrast to the ability of these VHH to bind RSC3 this argues against their being similar to VRC01. At the nucleotide level the B9 and A14 precursor Vg shares 69.83% identity with VH1-2*02 as while B9 and A14 are 68.84 and 68.73% identical respectively. The B21 precursor shares 45% nucleotide identity with VH1-2*02 and B21 is more dissimilar with only 38.75% identity. The closest human V gene to all three VHH precursors is VH3-23*04: B9 and A14 precursor Vg shares 88.19% and 82.81% nucleotide identity with VH3-23*04. While the mature B9 and A14 both share 85.26% V gene identity with VH3-23*04 and 74.51% and 74.00% J gene identity with JH5*01 respectively. The B21 precursor Vu shares 78.35% with VH3-23*04 but B21 has a much lower identity to any human V gene, the highest being to 81.05 to V3-66*02 and sharing 83.33% J gene identity with J4*02.

### Mutation of B9 VHH improves affinity, potency and breadth

**Figure 3:**
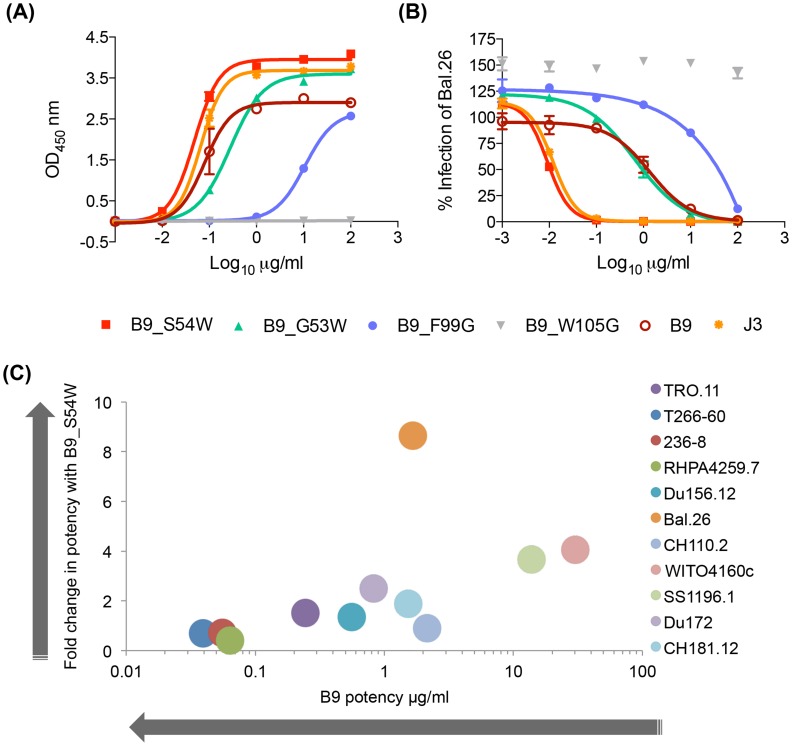
VHH mutation alters affinity, potency and breadth of neutralization. (A) VHH binding to clade B gp120 Bal.26 in ELISA detected by their C-terminal Myc tag. (B) VHH neutralization of Bal.26 HIV pseudovirus in the TZM-bl assay. Mutant VHH were generated by site-directed mutagenesis as detailed in the Materials and Methods. (C) The fold change in IC50 µg/ml values for B9 S54W relative to B9, indicating the increase potency of the mutant are plotted on the Y-axis. The IC50 µg/ml values for B9 against each virus in the legend are plotted on the X-axis, indicating the baseline potency of B9. All assays were carried out in duplicate and error bars represent standard deviation. These data are representative of at least three independent experiments.

Despite IC50 neutralization potency for some viruses as low as 0.02 µg/ml, we hypothesized that the affinity of these VHH could be improved either by additional affinity maturation in the llama or artificially by *in vitro* mutations of residues predicted to be involved in the interaction with Env. Because there are no known D genes for *Lama glama*, it has not been possible to identify a germ line sequence corresponding to the CDR3 loop of these antibodies, consequentially nor is it possible to calculate the amount of affinity maturation within this canonical antibody-antigen contact site. However, the presence of two aromatic residues at positions 99 and 105 in both A14 and B9 suggest potential Env contacts. Therefore, both these residues in turn were mutated to glycine to evaluate their relative contributions to the antibody-Env interaction. B9 F99G has a reduced ability to bind gp120 (Fig. 3A) and also a log-fold decrease in neutralization potency (Fig. 3B). The W105A mutation in B9 completely removes binding and neutralization function. Thus these residues are preferred and required for function respectively. Given the shared ability of A14/B9 and the VRC01-like antibodies to bind RSC3, we postulated that the structure-based insertion of an additional aromatic residue prior to CDR2 used to improve the affinity of the VCR01-like NIH45-46 antibody [38] could boost the affinity of A14/B9. The co-crystal structure of NIH45-46 with gp120 revealed that, despite extensive contacts between the two proteins, the hydrophobic patch on gp120 that is filled by an aromatic residue (phenylalanine 43) when CD4 is bound is not occupied when NIH45-46 binds Env. The insertion of a tryptophan at residue 54 in NIH45-46 resulted in improved affinity and median potency from 0.41 µg/ml to 0.04 µg/ml and expanded neutralization of six strains of HIV which are resistant to the parental mAb [38]. Thus a tryptophan was inserted into B9 in an attempt to improve its affinity for Env. Two B9 mutants were generated as, due to a longer CDR2 loop in B9 relative to the human antibody NIH45, it was not clear which residue in B9 corresponds with G54 in NIH45. Of these two B9 mutants, G53W bound to gp120_Bal.26_ with comparable affinity to B9 in ELISA whereas S54W bound more strongly than B9, with a higher maximum binding level, to an equivalent level of J3 (Fig. 3A).

Subsequently, the ability of the mutants to neutralize a pseudovirus bearing the Bal.26 envelope used in the ELISA was tested. In parallel to its wild type-like ability to bind gp120_Bal.26_, G53W neutralized with a similar IC50 of 2 µg/ml compared to 2.2 µg/ml for wild type B9 (Fig. 3B). The other tryptophan insertion pre-CDR2 mutant S54W instead showed increased neutralization potency with a twenty-fold increased IC50 value compared to wild type B9 (Fig. 3B). This shows that the increased affinity seen in the binding studies correlated to improved neutralization against a virus using the same Env protein. In addition, S54W neutralized TRJO4551.58 and CH038 strains, which are not neutralized by B9, with IC50 values of 37.3 and 32.9 µg/ml. While S54W showed more than a two-fold increase in IC50 against an additional three clade B, two clade C and one CRF BC strains (Du172, SS1196, WITO4160.33, DU156, CH181.12) the mutant VHH did not result in improved potency against the other clade B or CRF AG viruses tested (RHPA4259.7, TR0.11, T266-60, 236-8) (Fig. 3C, Table S3 in S1 Text). Furthermore, there was a trend for enhanced potency for S54W in strains that were less well neutralized by to B9 (Fig. 3C).

### Combining anti-CD4 VHH improves neutralization coverage

**Figure 4:**
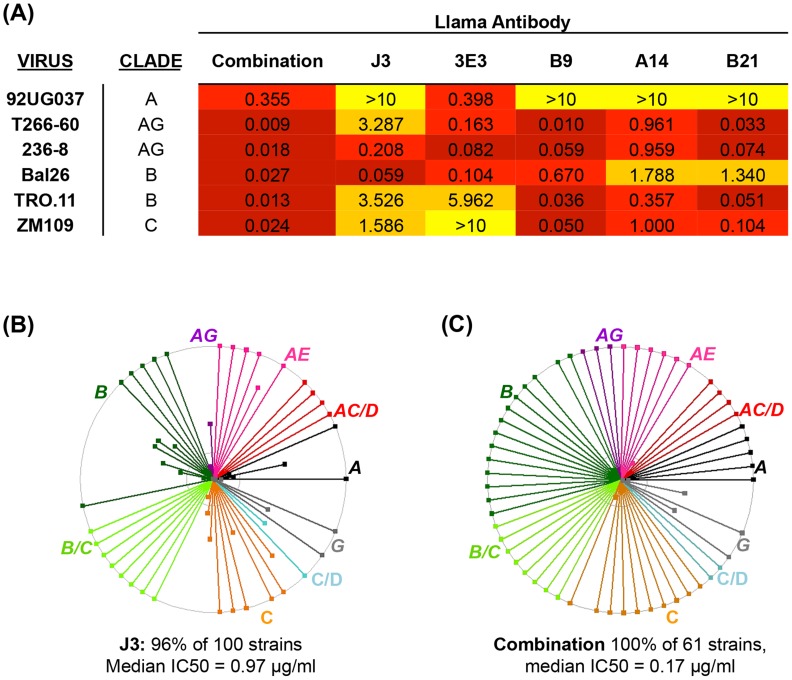
Combined breadth and potency of anti-CD4bs VHH. (A) IC50 values in µg/ml against the HIV strains indicated in the left-hand column on TZM-bl cells. VHH were titrated five-fold in duplicate both individually (from 10 µg/ml) and in combination with one another in the same plate for each virus. IC50 values of less than 0.1 are color-coded in dark red, those between 0.1 and 1 in red, those between 1 and 10 in orange and those above 10 in yellow. (B) IC50 values in µg/ml against each of the HIV strains indicated on the X-axis are shown for J3 or the combined VHH according to the color-coded legend. (C) Each spoke represents a strain of HIV neutralized by J3 or the combined VHH. Strains from different clades and CRF are color-coded according to the labels. The outer circle represents an IC50 of <1 µg/ml, the inner circle <5 µg/ml and the centre of the circle 50 µg/ml.

Over the last 5 years many broad and potent neutralizing mAbs have been identified [20], [33], [38], [39], [40], [41], [42], [43], [44] and it has been postulated that combining these mAb could provide protection against a wider range of strains whether by traditional passive transfer at much lower doses than previously imagined [11] or via novel gene transfer strategies [3]. We hypothesized that as human mAb can be successfully combined, without detrimental effects on their individual neutralization activities [19], A14, B9 and B21 identified in this study could also be combined with other VHH. These three VHH were mixed with J3 and 3E3 and then assayed for the ability to neutralize HIV in comparison to the same concentration (10 µg/ml) of each VHH in isolation. Viruses with differential susceptibilities to the five VHH were chosen from clades A, B, C and CRF_AG. For all six viruses the mix of five VHH resulted in an IC50 of improved or equivalent potency to the most potent of the five components (Fig. 4A). Therefore, these CD4 binding site VHH do not interfere with one another's function, thus we can extrapolate that the IC50 value for each virus in the panel for the mix of five would be equivalent to that of the most potent VHH against each particular virus (Fig. 4B). This represents an improvement over the use of J3 alone because, while J3 neutralizes 97% of this panel of 61 viruses, the combination neutralizes 100%, and for some viruses the potency of J3 is superseded by A14, B9, B21 or 3E3, resulting in a predicted median IC50 of 0.17 µg/ml for the combined antibodies (Fig. 4C).

### Affinity maturation of neutralizing VHH occurs in response to immunizations

Whether llamas produce neutralizing conventional antibodies against HIV in response to immunization is unknown, but it is plausible that the narrower HCAb are more easily elicited against recessed targets such as the CD4 binding site of HIV than conventional antibodies. Recent data have demonstrated that the anti-HIV potency of J3 is enhanced, when the VHH is presented in a full-length HCAb format, therefore, the neutralization activity of these VHH is not due to their being only the 15 kDa variable region [45]. However, whether or not HCAb can more easily be elicited than IgG does not alter one similarity between neutralizing antibodies derived from HIV-positive patients and J3, namely that their unmutated ancestors do not bind HIV Env [46], [47], [48], except when modified to do exactly that [49], [50], [51] or in one documented case of infection [52]. Reverting just three residues in J3 to germ line removes all ability to bind to either immunogen used to elicit the J3 parental HCAb [26]. Therefore, it is not clear how the antibody was elicited and whether the immunogen interacted with a rare naïve B cell bearing the unmutated ancestor or if the J3 B cell was only boosted by the HIV-based immunizations having been previously affinity matured in response to a different antigen encountered by the field-reared llamas.

To resolve this uncertainty, deep sequencing of VHH libraries was performed. This approach has previously provided insight into the development of VRC01-like antibodies in multiple HIV-positive donors [53], enabled analysis of multiple lineages with distinct specificities arising within an individual donor [54] and allowed in-depth analysis of the heavy chain V gene usage in Env immunized macaques [55]. Briefly, VHH from four immunized llamas and seven naïve llamas were amplified from the phagemid library by PCR using primers specific to the 5′ and 3′ conserved regions of the VHH and subjected to 454 sequencing. Firstly, we sequenced libraries from llamas 8 and 9 from which J3 and 3E3 were isolated respectively. Llamas 8 and 9 were both immunized with gp140 protein derived from HIV strains 92UG037 and CN54 [26], [31] (Table 1). Secondly, we sequenced libraries from llamas 1 and 3 described in this study, which produced the VHH A14, B9 and B21. Llama 1 and 3 libraries were generated at two separate time points: after the initial three sets of VLP-DNA immunizations and after four sequential gp140 protein boosts. In addition, seven naïve animals previously used to generate naïve VHH libraries from which antigen-specific VHH can be produced via *in vitro* affinity maturation [56] were sequenced as a control for the variation within the HCAb repertoire of the immunized llamas. All llamas used were genetically outbred and raised outdoors in contrast to laboratory animals often used for immunization studies.

**Figure 5:**
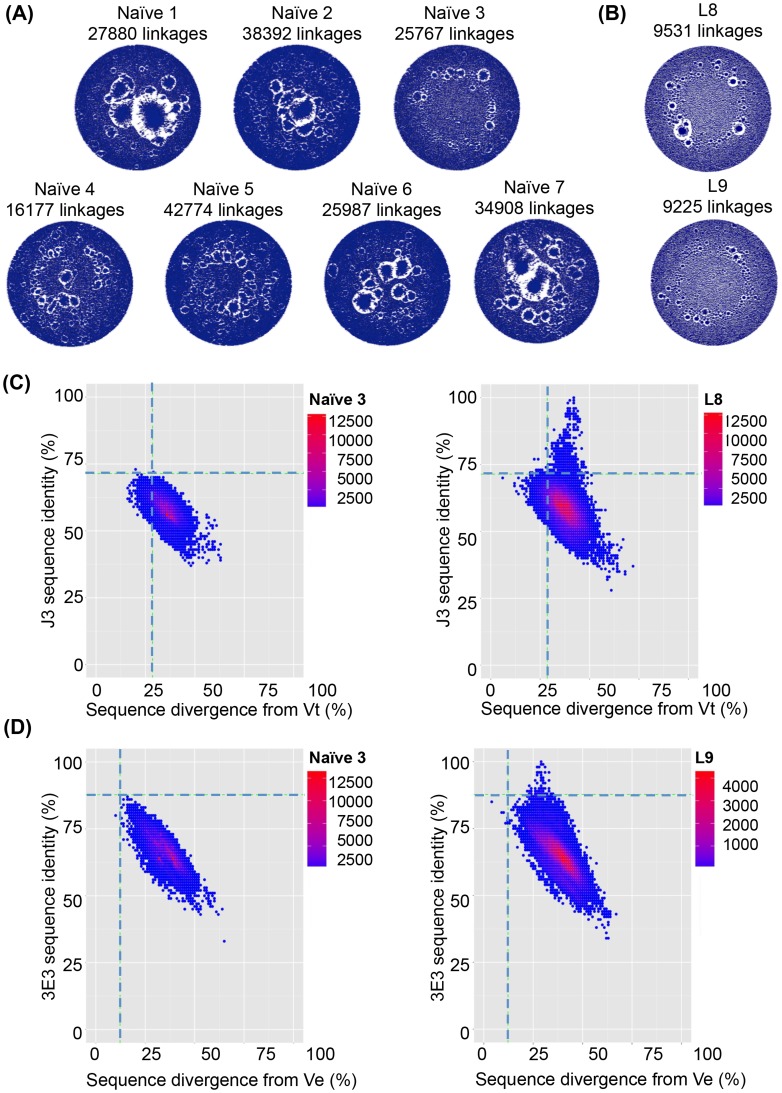
VHH repertoires of naïve and protein-immunized llamas. VHH sequences from immunized llamas 8 and 9 and seven naïve llamas were amplified from their respective phagemid libraries by PCR using primers specific to the 5′ and 3′ conserved regions of the VHH and subjected to 454 sequencing. (**A**) Unique sequences generated from the indicated llama phage library were used to build end-joining network diagrams with significantly more linkages (P = 0.001) in the naive llamas versus (**B**) immunized llamas. (**C**) Shared percentage identities with neutralizing VHH J3 and divergence from its inferred V gene Vt were calculated for all unique sequences from the control naïve llamas, and the J3-source llama 8. The left hand panel shows percentage identity for all sequences from naïve 3 plotted against divergence from Vt. The right panel shows percentage identity for all sequences from llama 8 plotted against divergence from Vt. The horizontal dotted line on each panel indicates the percentage identity shared by Vt and J3 and the vertical dotted line indicates the divergence of J3 from Vt, (**D**) Shared percentage identities with neutralizing VHH 3E3 and divergence from its inferred V gene Ve were calculated for all unique sequences from the control naïve llamas, and the 3E3-source llama 9. The left hand panel shows percentage identity for all sequences from naïve 3 plotted against divergence from Ve. The right panel shows percentage identity for all sequences from llama 9 plotted against divergence from Ve. The horizontal dotted line on each panel indicates the percentage identity shared by Ve and 3E3 and the vertical dotted line indicated the divergence of 3E3 from Ve.

**Figure 6:**
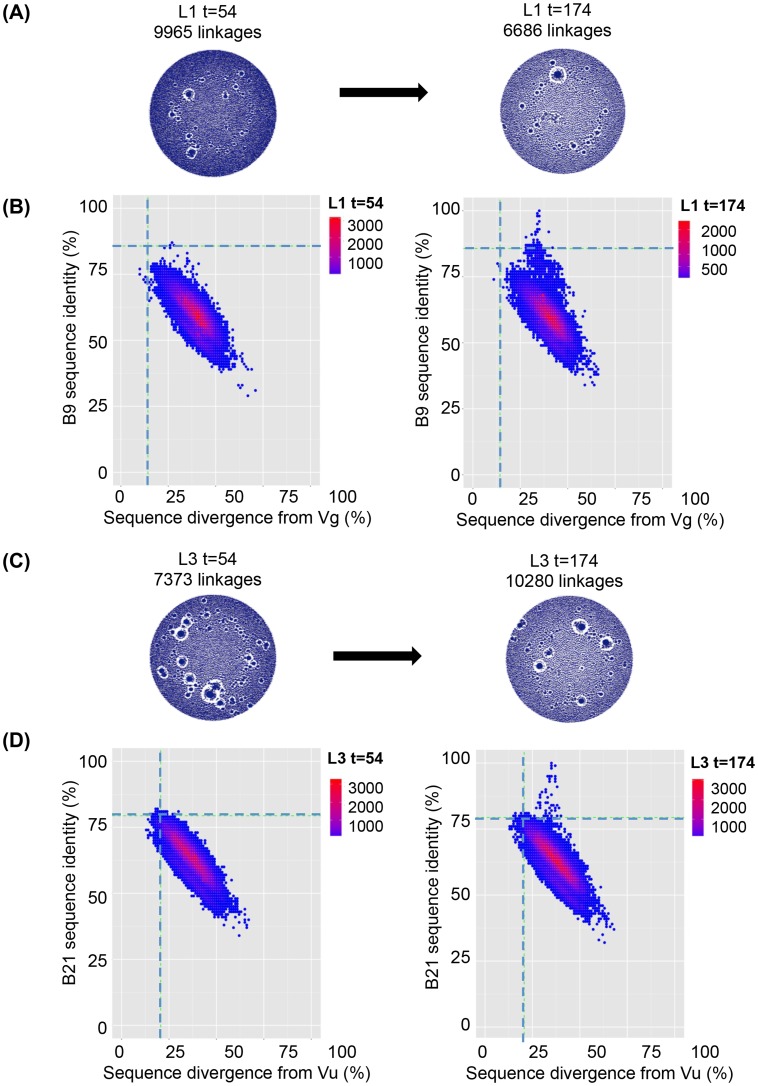
VHH repertoires post DNA/VLP priming and gp140 boosts. VHH sequences from immunized llama 1 at both t = 54 and t = 174 were amplified from their respective phagemid libraries by PCR using primers specific to the 5′ and 3′ conserved regions of the VHH and subjected to 454 sequencing. (**A**) Unique sequences generated from the llama 1 were used to build end-joining network diagrams with no significant difference in the number of network linkages between time points (**B**) Shared percentage identities with neutralizing VHH B9 its inferred V genes Vg were calculated for all unique sequences from both time points. Percentage identity with B9 for all sequences are plotted against divergence from Vg for t = 54 in the left hand panel and t = 174 in the right hand panel. (**C**) Unique sequences generated from the llama 3 were used to build end-joining network diagrams with no significant difference in the number of network linkages between time points (**D**). Shared percentage identities between neutralizing VHH B21 and its inferred V genes Vu were calculated for all unique sequences from both time points. Percentage identity with B21 for all sequences is plotted against divergence from Vu for t = 54 in the left hand panel and t = 174 in the right hand panel. In both (B) and (D) the horizontal dotted line on each panel indicates the percentage identity shared by the mature and germ line VHH and the vertical dotted line indicates the divergence of the mature VHH from its putative germ line precursor.

Two sequencing runs per VHH library were pooled resulting in 103677 to 213138 unique sequences per sample. Notably, the clonal structure of the VHH repertoire in the naïve animals differed to that in immunized animals (Table S4 in S1 Text). Network diagrams [57] (Figs. 5 and 6) were constructed whereby each point represents an individual sequence Any sequences differing by only one DNA base were connected by linkages. This results in large clusters within a network diagram for large clonal families and many individual points connected to only a few other points representing sequences with fewer clonal relatives. Thus, in a network diagram (normalized for number of sequences) with larger clusters, there are more linkages but fewer clonal families representing lower antibody diversity. This scenario is exemplified by the naïve llamas, whose networks had an average of 30269 linkages. In contrast, significantly fewer linkages (8843 linkages, p = 0.001) were seen in the immunized animals and only small clusters, representing an increase in the number of different clonal families as the VHH repertoire diversifies in response to the immunizations (Figs. 5A,B and 6A,C). The mean number of linkages per unique sequence was also found to be significantly larger for naive than immunized llamas, further illustrating the lower degree of divergence between the naive sequences (naïve: 0.22, immunized: 0.052 linkages per unique sequence, p<0.005, Student's t-test). The same result is obtained when normalizing the number of unique sequences by total reads per sample (naïve: 0.21, immunized: 0.050 linkages per unique sequence, p<0.005, Student's t-test).

However, due to the high level of VHH diversity, even in the immunized animals, it is not possible to identify clusters of clones specifically induced by immunization within the network diagrams. This is in contrast to readily identifiable dominant clusters observed in human B cell lymphoma patients [57] where one or few clonal lineages expand massively to dominate the network diagram. Therefore, to establish the relative frequency of neutralizing clones within the VHH repertoire, individual sequences were aligned to both the known neutralizing VHH sequences and their most likely germ line V gene sequences and the percentage identity to the neutralizing VHH plotted (Fig. 5C,D). The percentage nucleotide divergence (100 minus the percentage identity) from germ line and from the respective neutralizing VHH sequence for each of the five neutralizing VHH in each of the 13 llamas was examined. As some reads did not extend to the 5′ end of the V gene, percentage identity was calculated by dividing the total identity by the length of the query sequence. All reads however include the three complementarity determining regions (CDR). Notably, a population of sequences with identity to J3 greater than the germ line precursor (85.6%) is found only in immunized llama 8 which produced the J3 HCAb (population above the dotted line in Fig. 5C right-hand panel compared to naïve llama 3 sequences shown in left-hand panel). In addition none of the sequences obtained from any of the naïve llamas are more closely related to J3 than the germ line (85.6% identity) (Figure S4 in S1 Text).

For the other llama immunized in parallel (llama 9), the most broadly neutralizing VHH was 3E3 described herein. Again, a population of sequences with identity greater to 3E3 than its germ line precursor Ve (which differs to that of J3) was observed only for llama 9 (population above the dotted line in Fig. 5D right-hand panel compared to naïve llama 3 in the left-hand panel). Interestingly, llama 9 gave rise to no sequences with greater identity to J3 than germ line (Figure S4 in S1 Text) and llama 8 gave rise to no sequences closely related to 3E3. Therefore, the same immunogens and immunization protocol in two different animals gave rise to two separate clonal lineages of CD4-binding site broadly neutralizing HCAbs. Remarkably, both lineages have incurred a three-residue deletion in CDR2 during maturation suggesting the immunogens imposed constraints which resulted in a similar structural solution to high affinity binding that was achieved by different underlying sequences.

### DNA/VLP priming resulted in affinity maturation of VHH that resulted in binding but not to a sufficient level to enable neutralization

A limitation of the sequencing analysis of immunized llamas 8 and 9 described above is that they could be compared only to a cohort of seven naïve llamas and not to a pre-immunization time point as no samples from llamas 8 and 9 were available except after the final immunization. In contrast, llamas 1 and 3 immunized in the protocol described herein were sampled at two discrete time points: day 54 after the DNA/VLP immunizations and day 174 after four subsequent gp140 boosts. Serum samples from day 54 showed binding but no detectable neutralization activity (Figure S1 in S1 Text). In contrast, the serum samples from day 174 neutralized pseudoviruses from subtypes A, B and C and the neutralizing VHH A14, B9 and B21 were isolated from the phage library generated from lymphocytes obtained at day 174. VHH Networks showed no change in sequence interconnectivity between the two time points in either llama, and both time points showed a higher degree of immunefocusing on the encountered antigen as represented by a lower average number of network linkages as compared to the naïve llamas (Figs. 5A, B and 6A, C). Sequences from both time points were analysed to understand whether clones related to A14, B9 and B21 arose during the initial DNA/VLP immunization cycle at levels too low to result in sera neutralization and were then boosted by the protein immunizations or whether the protein alone was the antigen responsible for their affinity maturation (Fig. 6B and D). As seen for llamas 8 and 9, a population of sequences sharing a higher identity with the neutralizing VHH B9 or B21 than their germ lines was seen in both llamas after the protein immunizations at t = 174. However, no sequences from t = 54 in either llama had greater identity with the affinity matured VHH than the relevant germ line as indicated by the horizontal dotted line on each plot. The same pattern was seen when the closely related A14 VHH was used in place of B9 to analyse the sequences generated from llama 1 and both time points. Thus affinity maturation to a level that produced neutralizing activity was not achieved during DNA/VLP priming phase of the immunizations.

### Reverting V gene to germ line does not prevent B9, A14 and B21 function

**Figure 7:**
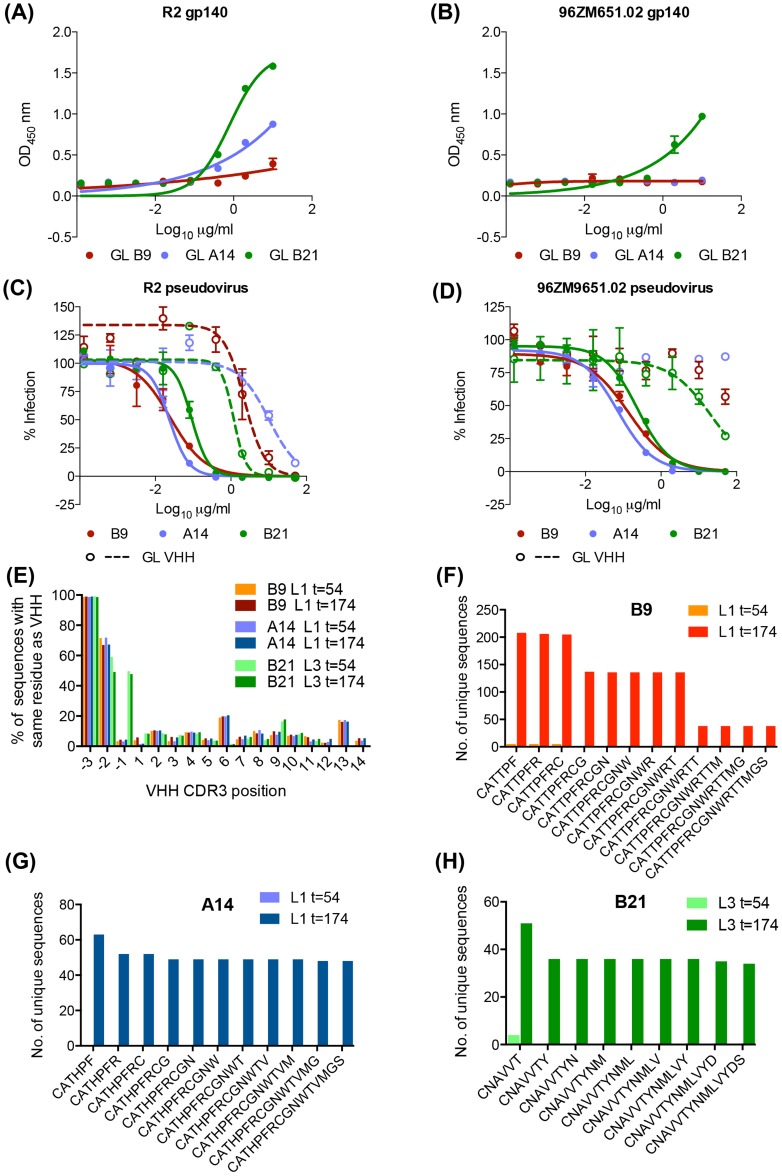
Anti-HIV activity of germ line V gene VHH. GL VHH comprising inferred germ line V gene paired with the mature VHH CDR3 and J region were produced. GL VHH binding to (**A**) clade B immunogen R2 gp140 and (**B**) clade C immunogen 96ZM9651.02 gp140 was assessed by ELISA as per the materials and methods. GL VHH neutralization of (**C**) R2 pseudovirus and (**D**) 96ZM651.02 pseudovirus was assessed in TZMbl assay as per the materials and methods. (**E**) Unique sequences from llamas 1 and 3 at both timepoints were filtered for B9, A14 and B21 germ line V gene usage (Vg and Vu) respectively. The y-axis shows the percentage of sequences within each subset with identical residues to each VHH at each individual CDR3 position (and the preceeding three V gene residues −1,−2,−3 = CAT/CNA) indicated on the x-axis. (**F, G, H**) The number of unique sequences within the subsets which share exact runs of CDR3 residues are plotted on the y-axis against runs of increasing CDR3 length on the x-axis for (**F**) B9, (**G**) A14 and (**H**) B21 at the time points indicated in the legend.

Despite representing two separate neutralizing lineages derived from two animals (Table 1) J3 and 3E3 have both incurred a three-residue deletion in CDR2 during maturation and the reversion of this mutation abrogates J3 function [26]. Similarly, re-insertion of the three germ line residues into 3E3 removed its ability to bind gp140 (Figure S2 in S1 Text). This suggests the immunogens imposed constraints resulting in a common structural mode of high affinity encoded by different sequences. To gain insight into which mutations incurred during affinity maturation were responsible for the A14, B9 and B21 function, germ line reverted VHH (GL VHH) were recombinantly produced. These comprised the relevant germ line V gene paired with the mature CDR3 and J sequence of each VHH (Figure S3 in S1 Text). Both GL B9 and A14 showed measurable binding to the R2 gp140 immunogen by ELISA, which was weaker than that seen for the unreverted VHH (Fig. 2A,B), but no binding to 96ZM651.20 gp140 (Fig. 7A,B). Thus the key mutation events required for Env binding are within the CDR3, which in the GL VHH is the same as in the matured VHH. GL B21 on the other hand bound to both immunogens (Fig. 7A,B) although to a lesser degree than B21 (Fig. 2A,B) which has additional mutations within the V gene. A similar pattern of neutralization activity for the GL VHH was seen, with GL B9 and A14 neutralizing R2 only and GL B21 neutralizing both autologous viruses (Fig. 7C,D) although in all cases the neutralization by the GL VHH was less potent than by the mature VHH. This raised the question of whether these GL VHH, which have a mature CDR3, occurred in the llamas prior to immunization or at t = 54 in addition to at t = 174 when the phagemid library from which the VHH were isolated was generated. Although no neutralization activity was observed at t = 54 it is theoretically possible these VHH could be present but at extremely low frequencies which would not result in neutralizing sera. However, as described above no sequences with greater identity to the affinity matured VHH than germ line were identified at t = 54 (Fig. 6). To further examine whether any clones similar to the GL VHH produced recombinantly were present at t = 54 additional analysis of the sequencing data was performed. Sequences were filtered into subsets, which shared V gene usage with each VHH. The CDR3 sequences of the subsets were then analyzed for the frequencies of residues that matched to those found in the mature CDR3 at the same position and the number of unique reads where sequential CDR3 residues (runs) matched to the mature CDR3 sequence. Total frequencies of matching residues at individual CDR3 positions did not vary substantially between time points for any of the VHH in each llama (Fig. 7E). However, no unique sequences were found at t = 54 which matched the mature CDR3 for more than the initial 5,1, or 3 CDR3 residues for B9, A14 and B21 respectively (Fig. 7F,G,H) following the CAR/CNA residues found at the end of each V gene. In contrast multiple copies of sequences with fully matching CDR3s were found for all VHH in the t = 174 subsets. Thus the GL VHH tested in this study which both bind the R2 immunogen and neutralize the corresponding virus were not present at t = 54 at detectable level by 454 sequencing and it is therefore likely that they were absent or extremely rare at t = 0.

## Discussion

This study has demonstrated that broadly HIV neutralizing llama HCAb can be reproducibly elicited by immunization (Fig. 1). Despite diverse immunization protocols, the best HCAb isolated by direct screening of VHH for neutralization breadth all target the CD4-binding site of HIV Env (Fig. 2) and those in most recent immunization study can bind to the CD4-binding site focusing gp120 mutant RSC3. Notably, the serum binding titres for RSC3 and RSC3delta are highly comparable and thus the sera responses of these llamas are not dominated by RSC3-specific anti-CD4-binding site clones, and the neutralizing VHH described herein are minority variants of the total response, in agreement with the weak neutralization titres observed (Figure S1 in S1 Text) and the low overall frequency of sequences belonging to these lineages (Fig. 6). It may be that only CD4-binding site specific lineages were isolated due to easier elicitation of such specificities due to the site's conservation within the unstabilised gp140 protein immunogens used (unlike trimer-specific epitopes) or due to the preference of the single-headed HCAbs for long CDR3 protrusions which can effectively bind into recessed canyons such as the CD4-binding site. It must also be noted that only one broadly neutralizing lineage in each animal was identified from 5000 clones and other specificities may have been found by screening more clones. In addition, although the screening was performed on phagemid libraries derived from llama PBMCs using primers designed to amplify all known VHH families (T Verrips, unpublished data) bias may have been introduced by this process if certain sequences were less preferentially incorporated in to the library. Similarly, some VHH may not have been well expressed in the bacterial system, preventing them from being identified by the robotic screening.

Interestingly the fine specificities of CD4 binding vary between clones resulting in the ability of each clonal lineage of VHH to effectively neutralize some strains of HIV which are resistant to the other VHH (Fig. 1). A14, B9 and B21 are highly potent against representatives from HIV strains circulating in certain geographical areas that J3 does not target as efficiently. We have demonstrated that combining five of these VHH results in superior neutralization breadth compared to that of any of the VHH used in isolation (Fig. 4). This concurs with studies of human antibodies targeting independent epitopes [19]. However, all five VHH target the CD4 binding site, nevertheless, they can be used in combination to result in neutralization potency equivalent that of to the most potent VHH. This suggests the differences in affinity of each VHH for any particular virus are great enough to prevent the less potent VHH occupying the CD4 binding site resulting in less potent neutralization. That VHH elicited by immunizations which target the same site on Env can be used in combination without compromising neutralization potency is encouraging for vaccine design studies. It indicates that the co-induction of a variety of antibodies targeting the same site is not detrimental to neutralization function in itself, although how this can be achieved by immunization remains to be determined. Since the combination of these five anti-CD4 binding site VHH neutralized 100% of strains tested with an IC50 of less than 0.2 µg/ml (Fig. 4), their combined use in a topical anti-HIV microbicide [28] could provide a higher barrier to infection and viral escape than the use of any one VHH individually. It has previously been shown that virus escape occurs from passively transferred human antibodies used to treat infection when three or less are used in combination whereas no escape was seen from a combination of five antibodies [58]. How significant escape would be in the setting of chronic microbicide use remains to be determined but the use of multiple VHH targeting the same site in different ways should reduce the risk and provide a higher barrier to transmission.

We have also shown that the VHH described can be improved in terms of potency and breadth via site-directed mutagenesis. The residues altered were selected based on the observation that the three VHH described herein, B9, A14 and B21, bind to the RSC3 probe that was used to isolate the VRC01-class of human CD4 binding site broadly neutralizing antibodies (Fig. 2) and that previously the introduction of an aromatic residue into the VRC01-like antibody NIH45-46 resulted in increased breadth and potency [38]. The introduction of an aromatic in the mutant B9S54W also resulted in increased potency and breadth (Fig. 3) despite the lack of significant sequence homology between VRC01-like antibodies and B9, A14 and B21 (compounded by the lack of a light chain which contributes to the VRC01-Env interface). Thus, it cannot be concluded that these VHH are VRC01-like or that these immunogens can elicit such antibodies in humans. It would be of interest to undertake structural studies to establish whether there are any structural similarities.

While the overall frequency of A14, B9 and B21-like sequences is low there is a clear expansion of these lineages after the animals received four gp140 protein boosts (Fig. 6). This low frequency agrees with the weak neutralization breadth seen for the t = 174 serum samples (Figure S1 in S1 Text). In contrast, the deep sequencing analysis of VHH present at t = 54 shows no over-representation of the sequences which share higher levels of identity with the neutralizing VHH than the germ line (Fig. 6). This suggests that the low-level maturation achieved during the DNA/VLP immunizations was not sufficient for neutralization or the definition of these clonal lineages. Interestingly, A14, B9 and B21 GL VHH (with the V gene reverted to germ line but mature DJ regions) were all able to bind the R2 immunogen and neutralize the homologous virus (Fig. 7) but only B21 GL could bind the both immunogens and neutralize both viruses. This difference in binding ability to the two immunogens suggests the R2 immunogen may have driven the earlier stages of affinity maturation of this lineage in llama 1, which resulted in clones that could also recognize 96ZM651.02 gp140 as the immune response progressed and the lineage developed breadth via mutations in the V gene region. That these germ line reverted VHH could bind and neutralize at first appears to contradict the lack of neutralization activity and the inability to identify clones belonging to each lineage at t = 54. However, further sequencing analysis established that these germ line chimera were not present at the earlier time point, as no sequences with the fully mature DJ regions paired to the relevant germ line V genes were detected at t = 54 whereas between 50 and 250 copies of such clones were present at t = 174. Thus we can conclude that mutations occurred in the CDR3s during the protein immunization phase resulting in neutralization ability, alongside additional mutations in the V gene which are required for breadth at least in the A14/B9 lineage. The germ line chimeras do however demonstrate that these VHH require only mutations within their CDR3 for neutralization activity. This is in agreement with the finding that the F99G and W105G substitutions in the CDR3 of B9 diminish and destroy its anti-HIV activity respectively (Fig. 3A,B). This is in contrast with the fact that J3 and 3E3 require a deletion within the V gene which shortens the CDR2 loop by 3 residues [26], (Figure S2 in S1 Text) to bind and neutralize HIV Env.

Notably, the global incidence of A14, B9 and B21-like sequences is low within even the t = 174, however this is across total VHH from whole peripheral blood, not just those specific for these immunogens because no panning enrichment was performed with the phagemid library prior to neutralization screening. This greatly increases the signal-to-noise ratio in the deep sequencing analysis and thus it is not possible to identify which clones are immunogen-specific and analyse these as a subset. This is because a primary sequence alone does not allow us to predict function unless the sequence is highly similar to a characterized clone. Therefore, future studies should involve an immunogen-specific flow cytometry selection of the immungen-specific llama B cells prior to generating the library to more easily identify clusters of immunogen-specific VHH. Such studies may also provide insight into whether the high level of divergence from germ line (relative to human immunization studies) seen in these clones is standard in the HCAb response to immunization. That HCAb memory B cells may be more mutated from germ line relative to conventional IgG B cells is not entirely unanticipated. Naïve HCAbs inherently start from a less diverse paratope repertoire as they do not combine a heavy and light chain, both derived from VDJ recombination. Therefore, in order to successfully advance through successive rounds of affinity maturation and compete for immunogen with the conventional antibodies, llamas also produce (estimated at 70% of total IgG) the HCAbs that may undergo additional mutations. In addition, the mutation process is inherently random, and deleterious (stop codons etc.). In a normal B cell a deleterious mutation in either chain will prevent expansion of that lineage, whereas in a HCAb B cell a successful heavy chain does not require a functioning light chain to proceed.

It is significant that parallel immunization of llamas 8 and 9 gave rise to two separate clonal lineages of CD4 binding site broadly neutralizing HCAbs which have both incurred a three residue deletion in CDR2 during maturation. This finding indicates that the immunogens imposed constraints, which resulted in a similar structural solution to high affinity binding achieved by different sequences. This has implications for the use of deep sequencing analysis of immunization studies: if the sequence of J3 had been used as a reference to filter the sequences from llama 9 (without prior screening of VHH from llama 9), no J3-like antibodies would have been found, 3E3 would not have been identified and it would have falsely appeared that the immunization of llama 9 had failed. These findings are consistent with the observation that HIV Env immunization activates a highly polyclonal B cell response of substantial genetic diversity in NHP [55]. This is in contrast to the well-characterized VRC01-like family of CD4-binding site antibodies identified in multiple HIV-positive donors, which share both a heavy chain V gene precursor and unusual features at a DNA sequence level which could allow sequencing-based identification of other similar antibodies in distinct patients [53]. Thus, this study has implications for analysis of human vaccine studies, as in addition to searching for defined lineages it is worthwhile to perform functional analysis of monoclonal antibodies that may have found new structural solutions to high affinity binding which cannot be discerned from DNA sequence alone.

A critical implication of this work for the field of HIV vaccine design is the observation that the most potent and broad individual anti-HIV VHH, J3, was elicited in response to the gp140 immunogens used. It has been suggested that the extensive affinity maturation of antibodies and HCAbs which neutralize HIV could be the result of prior affinity maturation of clones to a non-HIV antigen. Hypothetically, such a B cell clonal lineage could have a greater affinity for Env than any germ line B cell and could either be boosted or further affinity matured in response to Env immunization. Deep sequencing analysis has shown that this is highly unlikely in the case of J3 as no sequences were generated by deep sequencing of the seven naïve llamas or three other immunized llamas that share greater identity with J3 than its putative germ line V gene. Paradoxically, it remains the case that even minimal mutation towards the J3 germ line V gene abolishes the ability to bind either immunogen used [26]; the reintroduction of the three germ line CDR2 residues renders the VHH incapable of binding Env. However, it must be noted that the llama immunization model is not only an animal model but one resulting in a subtype of antibodies not produced in humans. Furthermore, the neutralizing HCAb induced occurs in the llamas at a low frequency that does not result in broadly neutralizing sera, the goal for a protective HIV vaccine. However, this model has allowed us to examine four HIV broadly neutralizing clonal lineages induced by vaccination, which has not been possible in other animal models to date, and highlights the many challenges of evaluating immunization studies with deep sequencing of antibody variable regions.

## Materials and Methods

### Ethics statement

This study was performed in strict accordance with the Dutch Experiments on Animals Act 1997. In accordance with article 18 of the Act, the protocol was assessed and approved by the Animal Ethics Committee of Utrecht University (permit number: DEC#2007.III.01.013). All efforts were made to minimize discomfort related to immunizations and blood sampling. The animal welfare officers of Utrecht University checked the mandatory administration and supervised the proper conduct of procedures and the well-being of the llamas that were used.

### Immunization of *L. glama* and construction of the VHH phage library

Llamas 1 and 3 were immunized via intramuscular injection of plasmid DNA encoding R2 and 96ZM gp160 in PBS (7.5 mg of DNA) with or without virus like particles bearing R2 and 96ZM envelope proteins in PBS (protein content 50 µg). Subsequently additional immunizations were administered with intramuscular injection of ZM96 & R2 gp140 protein (50 µg each) in a freshly prepared 4.5-ml water in oil emulsion prepared by vigorously mixing 2 volumes of antigen with 2.5 volumes of the adjuvant Stimune (CEDI Diagnostics). Immunizations and VHH library construction were performed as described previously (De Haard et al., 2005). In brief, the llamas received one dose of DNA alone, then two doses of DNA combined with VLP, followed by four doses of protein in adjuvant as per Table S1 in S1 Text. The anti-Env immune response in sera was verified via neutralization of three viruses in TZM-bl cells (Figure S1 in S1 Text). Total RNA was isolated from between 120 and 150 million peripheral blood lymphocytes (PBMC) collected after immunization (on day 54 and 174) and cDNA was prepared. The VHH repertoire was amplified and cloned into the pCAD50 phagemid vector. To obtain recombinant bacteriophages expressing the VHH as fusion proteins with the M13 bacteriophage gene III product, transformed TG1 E. coli cells were grown to logarithmic phase and then infected with helper phage M13KO7. The phage particles were precipitated with polyethylene glycol.

### VLP and DNA immunogens

Codon optimized and c-terminal truncated (aa714–856, HXB2 numbering) R2 and 96ZM651 gp145 genes were synthesized (GeneArt) and cloned into pcDNA3.1 using NheI and PmeI restriction sites and NheI and XhoI respectively. DNA for immunizations was prepared using the Qiagen EndoFree Plasmid Giga Kit according to the manufacturer's instructions. A Giga-prep was performed to obtain at least 60 mg of DNA. 200 µg of R2 virus like particles (VLPs) and 200 µg of 96ZM VLPs were made. Pseudotyped VLPs for immunization purposes were produced in 293F cells by transient transfected with a codon-optimized, Rev-independent gene for Gag(IIIB) [59] and the respective envelope encoding plasmid in a ratio of 2∶1. VLPs were harvested 72 h post-transfection, cleared by centrifugation at 3000 g for 15 min, loaded onto a 30% sucrose in PBS cushion (5 ml for 30 ml of supernatant) and ultra-centrifuged at 100,000 g for 2 h. The pellet was resuspended overnight in PBS and stored at −80°C. For the quantification of incorporated envelope protein an ELISA was used. Each subsequent ELISA washing step was carried out with 200 µl PBS+0.05% Tween20 and each incubation was done for one hour at room temperature. A Clear 96-well MaxiSorp plates (NUNC) were coated overnight with 1 µg/ml of antibody 5F3 (Polymun). Plates were blocked using 10% fetal calf sera in PBS+0.05% Tween20 followed by three washings. Pseudotyped VLPs were denatured in the presence of 0.5% Triton-X for 1 h and the VLPs as well as recombinant gp140 standard protein were added in serial dilution followed by incubation. After three additional washing steps 50 µl of a 1∶1000 dilution of antibody MH23 (NIBSC) was added and incubated. The plate was washed three times before adding 0.65 µg/ml anti-mouse horseradish peroxidase–conjugated Ab (Dako) followed by incubation. After six washes TMB ELISA substrate was added, and the plates were incubated until standard proteins were visible. Purified gp140 from R2 and from 96ZM were mixed with Stimune commercially available Stimune adjuvant (CEDI Diagnostics, Lelystad, The Netherlands).

### Recombinant HIV-1 Env proteins

Recombinant trimeric gp140 from HIV-1 92UG037 (clade A) for ELISAs was provided by S. Jeffs (Imperial College London, London, England, UK). Recombinant D368R and wild-type monomeric gp120 from HIV-1 YU2 (clade B) and recombinant RSC3 and RSC3Δ37 for ELISAs were provided by J. Mascola (National Institutes of Health [NIH], Bethesda, MD). Recombinant gp41 from HIV-1 IIIB, recombinant trimeric gp140 from HIV-1 CN54 and BX08 were obtained from the CFAR, NIBSC and were donated by Immunodiagnostics and Polymun Scientific, respectively.

### Cells

TZM-bl cells (Derdeyn et al., 2000; Wei et al., 2002; Li et al., 2005) were obtained through the NIH AIDS Research and Reference Reagent Program from J.C. Kappes (University of Alabama at Birmingham, Birmingham, AL), X. Wu (NIAID, NIH), and Tranzyme, Inc. and cultured in Dulbecco's modified Eagle medium (Invitrogen) containing 10% (vol/vol) FCS.

### Viruses

Pseudoviruses were generated from two separate plasmids, one encoding a full length HIV virus with a defective *env* and the other encoding a functional *env*. This results in non-replication competent HIV progeny viruses that undergo only one cycle of infection, which is sufficient to test the ability of an antibody to inhibit HIV entry into cells. HIV-1 IIIB was propagated in H9 cells all other replication-competent virus stocks were prepared from HIV-1 molecular clones by transfection of 293T cells. HIV-1 IIIB (ARP101) was obtained from the CFAR, NIBSC. IIIB was donated by R. Gallo and M. Popovic (University of Maryland School of Medicine, Baltimore, MD). HIV-1 Env pseudotyped viruses were produced in 293T cells by co-transfection with the pSG3Δenv plasmid (Kirchherr et al., 2007). The subtype B and C HIV-1 Reference Panels of Env Clones (Li et al., 2005, 2006) were obtained through the NIH AIDS Research and Reference Reagent Program, Division of AIDS, National Institute of Allergy and Infectious Diseases (NIAID), NIH. HIV-1 subtype CRF07_BC Gp160 clones, subtype CRF02_AG Gp160 clones (263-8, T278-50, and T266-60), and the 92UG037, 93MW965.26, and 96ZM651.02 Gp160 clones were provided by D. Montefiori (Duke University Medical Center, Durham, NC) through the Comprehensive Antibody Vaccine Immune Monitoring Consortium (CA2 VIMC) as part of the CAVD. All additional pseudoviruses were produced at the CAVD Preclinical Neutralizing Antibody Core laboratory.

### Serum neutralization

Serum samples were heat-inactivated to destroy complement by incubation at 56°C for 1 h before use in neutralization assays. Threefold serial dilutions of llama sera were then tested, starting at a 1∶5 dilution in the 96-well plate the TZM-bl cell-based assay developed by Derdeyn et al. (2000), Wei et al. (2002), and Li et al. (2005), with Bright-Glo luciferase reagent (Promega) using a Pherastar plate reader (BMG Labtech).

### Isolation of anti–HIV-1 VHH through robotic direct HIV-1 neutralization screening

Phages expressing the cloned VHH repertoire were plated onto agar containing 100 µg/ml ampicillin and 2% syncytial stain (1 g methylene blue and 0.33 g basic fuchsin in 200 ml methanol). Individual clones were picked using a Norgren CP7200 colony picker (RapidPick; Hudson Robotics) into 384-well master plates. 6144 individual clones were expressed in TG1 E. coli cells in a 384-well plate format. Each clone was expressed in 150 µl of 2× TY medium containing 100 µg/ml ampicillin and 0.1% glucose, followed by induction of VHH production with 0.1 mM isopropyl-β-dthiogalactopyranoside. Bacterial pellets were frozen at -80°C for a minimum of 1 h and then thawed and resuspended in PBS. The periplasmic extract from each well was separated from bacterial debris by filtration through a 0.45-µM polyvinylidene fluoride membrane and screened for the ability to neutralize HIV-1. To enable high-throughput screening and characterization of VHH, neutralization was measured using 50 50% tissue culture infective doses of virus in a 384-well plate adaption of the 96-well plate the TZM-bl cell-based assay developed by Derdeyn et al. (2000), Wei et al. (2002), and Li et al. (2005), with Bright-Glo luciferase reagent (Promega) using a Pherastar plate reader (BMG Labtech). DNA from the individual VHH that neutralized any tier 2/3 viruses to <20% seen with control was purified, sequenced, and recloned into the pCAD51 expression vector followed by transformation into TG1 cells for purification and further characterization.

### VHH purification and neutralization characterization

Expression from the pCAD51 vector incorporates a 6-His and a c-Myc tag to the C terminus of the VHH and removes the bacteriophage gene III product. The VHH were purified by means of the attached His tag using TALON Metal Affinity Resin (Takara Bio Inc.). Mutagenesis of VHH DNA was achieved using the QuikChange site-directed mutagenesis kit (Agilent Technologies) according to the manufacturer's instructions. Mutant VHH protein was expressed and purified as described above. The neutralization activity of the VHH was assayed in duplicate/triplicate at either University College London or VIMC laboratories. No virus inactivation was observed with a negative control VHH with no specificity for HIV was used as control (De Haard et al. 2005). or with a pseudovirus bearing a mouse leukemia virus Env. VHH IC50 titers were calculated using the XLFit4 software (IDBS) or the Labkey Neutralizing Antibody Tool (Piehler et al., 2011). Cran R radial plots were used to display the inverse IC50 values (Fig. 1).

### ELISAs

Clear 96-well MaxiSorp plates (Thermo Fisher Scientific) were coated overnight with 2 µg/ml of recombinant Env. Plates were blocked using 5% milk powder in TBS. Serial dilutions in TBS supplemented with 0.05% Tween (TBS-T) containing 1% milk powder (TMT) of the VHH to be assayed and of a negative control VHH were then added to the plates in triplicate wells, and the plates were incubated at room temperature for 1 h and subsequently washed four times with TBS-T. The wells were then incubated with 0.5 µg/ml mouse anti–c-Myc–horseradish peroxidase–conjugated Ab (Roche) in TMT for 1 h at room temperature. After six washes with TBS-T, TMB ELISA substrate (Thermo Fisher Scientific) was added, and the plates were incubated at 37°C for 0.5 h. Absorbance at 450 nm was detected, and background-subtracted data were plotted against VHH concentration.

### Sequencing

Two technical replicates were run for each sample and the corresponding sequence datasets were merged. Any reads that were identical sub-sequences of other reads were removed and the read count for the longer sequences adjusted accordingly. Raw MiSeq reads were filtered for base quality (median>32) using the QUASR program (http://sourceforge.net/projects/quasr/). Overlap between forward and reverse reads, where no nucleotide mismatches are allowed. MiSeq forward and reverse reads were merged together if they contained identical overlapping region of>65 bp, or otherwise discarded. No nucleotide mismatches are allowed in the overlap regions, as this would be indicative of sequencing error or recombination. Primer sequences were trimmed from the reads, and sequences retained for analysis only if both primer sequences were identified with 100% match. Non-immunoglobulin sequences were removed and only reads with significant similarity to reference Llama IgHV and IgHJ genes using BLAST (Altschul et al., 1990) were retained (10-10, 10-3 e-value threshold respectively due to differences in gene length). Reads were retained if they contained complete open reading frames (without stop codons). Non-functional BCRs, PCR error or recombination may lead to the artificial introduction of stop codons. Length filter: between 150 and 320 nucleotides. If recombination was to occur, this filter would remove reads that had significant changes in their length. Graphs for each sample were generated using the igraph R library [60] with nodes corresponding to individual read sequences and linkages between nodes whose sequences differed by at most one nucleotide. Node sizes were proportional to the number of observed reads for each sequence. Sequence similarities were calculated by performing BLASTX [61] searches on read sequences against a database of known V gene products and reference sequences (J3, 3E3, A14, B9 and B21), counting insertions and deletions in the alignments as non-identical residues. Divergence was calculated as (100 – percentage sequence similarity). Divergence plots were generated using the ggplot2 R library [62]. Custom Perl and R scripts were used throughout to parse and analyse the sequence datasets (R Development Core Team, 2008 http://www.R-project.org).

## Supporting Information

S1 Text **Table S1: Immunization Schedule.** Llamas 1 and 3 were immunized via intramuscular injection as indicated in the table. Protein injections were in a freshly prepared 4.5-ml water in oil emulsion prepared by vigorously mixing 2 vol U of antigen with 2.5 vol U of the adjuvant Stimune (CEDI Diagnostics); **Table S2: IC50 against 77 viruses.** VHH were titrated threefold from 50 µg/ml and incubated with the indicated pseudoviruses on TZM-bl assay as described in the materials and methods. No virus inactivation was observed with a negative control VHH or with a pseudovirus bearing a mouse leukemia virus Env. VHH IC50 titers were calculated using the XLFit4 software (IDBS) or the Labkey Neutralizing Antibody Tool (Piehler et al., 2011). Potent neutralization (IC50<1 µg/ml) is color-coded red, intermediate neutralization (1–10 µg/ml) is color-coded yellow and weak neutralization (10–50 µg/ml) is color-coded green, non neutralization (>50 µg/ml) is colour-coded white, strains that weren't tested for particular VHH are indicated by a black dot; **Table S3. IC50 values for B9 VHH and S54W mutant against nine viruses.** The S54W mutant was generated by site-directed mutagenesis as described in the materials and methods. VHH were titrated threefold from 50 µg/ml and incubated with the indicated pseudoviruses on TZM-bl assay. VHH IC50 titers were calculated using the XLFit4 software (IDBS). Highly potent neutralization (IC50 <0.1 µg/ml) is color-coded dark red, potent neutralization (0.1–1 µg/ml) is color-coded red, intermediate neutralization (1–10 µg/ml) is color-coded yellow, weak neutralization (10–50 µg/ml) is color-coded green; **Table S4: Statistical analysis of sequence variation between immunized and naïve llamas.** The mean cluster size was found to be significantly larger for naive than immunized llamas. When considering only non-singleton clusters (i.e. clusters of sequences with 2 or more members) the average cluster size was also considerably larger for the naive llamas. The mean number of reads per unique sequence was also higher in the naive compared to the immunized samples highlighting the greater sequence diversity in the immunized samples. This was despite the total number of reads not varying significantly between either set of samples and further reinforced by the immunized animals generating significantly higher numbers of unique sequences per sample; **Figure S1: Post-immune sera anti-HIV activity.** (A) Threefold serial dilutions of llama sera were tested against the indicated pseudoviruses, starting at a 1∶5 dilution in the 96-well plate the TZM-bl cell-based assay as described in the materials and methods. (B) Threefold serial dilutions of llama sera were tested against the indicated immunogens, starting at a 1∶20 dilution. Binding was detected with HRP-conujugated anti-llama antibody. Serum samples were heat-inactivated to destroy complement by incubation at 56°C for 1 h before use; **Figure S2: VHH binding to HIV Env proteins.** VHH binding to (**A**) clade B gp140 BX08, (**B**) clade B gp41 IIIB and (**C**) clade A gp140 92UG037 was assessed by ELISA as described in the Materials and Methods. The positive control for gp140 binding was J3 [26] and that for gp41 binding 2H10 [32]; **Figure S3. VHH sequence alignments.** Alignment of A14, B9, B21, 3E3, their germ line V genes and human V gene VH3-23*04 and VH1-2*02; **Figure S4. Shared sequence identity with J3 relative to divergence from germ line for all naïve and immunized llamas.** Shared percentage identities with neutralizing VHH J3 and divergence from its inferred V gene Vt were calculated for all unique sequences from the seven control naïve llamas, and the four immunized llama including the J3-source llama 8. Each panel shows percentage identity for all sequences from the indicated llama plotted against divergence from Vt.
(PDF)
