# Supplementary material for: Molecular Evolution of Broadly Neutralizing Llama Antibodies to the CD4-Binding Site of HIV-1
Source: PLoS Pathog. 2014 Dec 18;10(12):e1004552. doi: 10.1371/journal.ppat.1004552 (PMC4270772; doi:10.1371/journal.ppat.1004552)
Supplement: S1 Text — Table S1: Immunization Schedule. Llamas 1 and 3 were immunized via intramuscular injection as indicated in the table. Protein injections were in a freshly prepared 4.5-ml water in oil emulsion prepared by vigorously mixing 2 vol U of antigen with 2.5 vol U of the adjuvant Stimune (CEDI Diagnostics); Table S2: IC50 against 77 viruses. VHH were titrated threefold from 50 µg/ml and incubated with the indicated pseudoviruses on TZM-bl assay as described in the materials and methods. No virus inactivation was observed with a negative control VHH or with a pseudovirus bearing a mouse leukemia virus Env. VHH IC50 titers were calculated using the XLFit4 software (IDBS) or the Labkey Neutralizing Antibody Tool (Piehler et al., 2011). Potent neutralization (IC50<1 µg/ml) is color-coded red, intermediate neutralization (1–10 µg/ml) is color-coded yellow and weak neutralization (10–50 µg/ml) is color-coded green, non neutralization (>50 µg/ml) is colour-coded white, strains that weren't tested for particular VHH are indicated by a black dot; Table S3. IC50 values for B9 VHH and S54W mutant against nine viruses. The S54W mutant was generated by site-directed mutagenesis as described in the materials and methods. VHH were titrated threefold from 50 µg/ml and incubated with the indicated pseudoviruses on TZM-bl assay. VHH IC50 titers were calculated using the XLFit4 software (IDBS). Highly potent neutralization (IC50 <0.1 µg/ml) is color-coded dark red, potent neutralization (0.1–1 µg/ml) is color-coded red, intermediate neutralization (1–10 µg/ml) is color-coded yellow, weak neutralization (10–50 µg/ml) is color-coded green; Table S4: Statistical analysis of sequence variation between immunized and naïve llamas. The mean cluster size was found to be significantly larger for naive than immunized llamas. When considering only non-singleton clusters (i.e. clusters of sequences with 2 or more members) the average cluster size was also considerably larger for the naive llamas. The mea [file ppat.1004552.s001.pdf]

SUPPLEMENTARY MATERIAL

Supplementary table 1: Immunization Schedule.
Llamas 1 and 3 were immunized via intramuscular injection as indicated in the table. Protein injections were in a freshly prepared 4.5-ml water in oil emulsion prepared by vigorously mixing 2 vol U of antigen with 2.5 vol U of the adjuvant Stimune (CEDI Diagnostics).

Day	Immunogen	Adjuvant	Sample
0	Intramuscular injection of plasmid DNA encoding R2 and 96ZM651.02 gp160 in PBS (7.5 mg of DNA)	No	10 ml blood
14	Intramuscular injection of plasmid DNA encoding R2 and 96ZM651.02 gp160 in PBS (7.5 mg of DNA)	No	No
28	Intramuscular injection of plasmid DNA in PBS (7.5 mg of DNA) and VLPs bearing R2 and 96ZM651.02 envelope proteins in PBS (protein content 50 µg)	No	10 ml blood
35	Intramuscular injection of plasmid DNA in PBS (7.5 mg of DNA) and VLPs bearing R2 and 96ZM651.02 envelope proteins in PBS (protein content 50 µg)	No	No
42	VLPs bearing R2 and 96ZM651.02 envelope proteins in PBS (protein content 50 µg)	No	10 ml blood
49	VLPs bearing R2 and 96ZM651.02 envelope proteins in PBS (protein content 50 µg)	No	No
54	No	n/a	**150 ml blood, from llamas 1 & 3, used for library generation**
131	Intramuscular injection of 96ZM651.02 and R2 gp140 protein (50 µg each)	Stimune	10 ml blood
145	Intramuscular injection of 96ZM651.02 and R2 gp140 protein (50 µg each)	Stimune	10 ml blood
159	Intramuscular injection of 96ZM651.02 gp140 and R2 protein (50 µg each)	Stimune	10 ml blood
166	Intramuscular injection of 96ZM651.02 and R2 gp140 protein (50 µg each)	Stimune	10 ml blood
174	No	n/a	**150 ml blood, from llamas 1 & 3, used for library generation**

Supplementary Table 2: IC50 against 77 viruses.

VHH were titrated threefold from 50 µg/ml and incubated with the indicated pseudoviruses on TZM-bl assay as described in the materials and methods. No virus inactivation was observed with a negative control VHH or with a pseudovirus bearing a mouse leukemia virus Env. VHH IC50 titers were calculated using the XLFit4 software (IDBS) or the Labkey Neutralizing Antibody Tool (Piehler et al., 2011). Potent neutralization (IC50 < 1µg/ml) is color-coded red, intermediate neutralization (1-10µg/ml) is color-coded yellow and weak neutralization (10-50 µg/ml) is color-coded green, non neutralization (>50 µg/ml) is colour-coded white, strains that weren't tested for particular VHH are indicated by a ●.

VIRUS:	CLADE	B9	B21	A14	3E3	J3
92UG037	A	2.52	10.02	0.56	0.03	0.33
MS208.A1	A	41.5	>50	>50	0.17	6.5
Q461.e2	A	>50	>50	47.2	0.31	●
Q23.17	A	0.04	0.09	0.28	3.04	7.73
Q842.d12	A	>50	>50	>50	0.13	0.57
3301.v1.c24	AC	<0.02	0.03	<0.02	0.18	0.97
6041.v3.c23	AC	1.32	2.17	1.31	<0.02	1.82
6540.v4.c1	AC	>50	>50	>50	>50	3.72
0815.v3.c3	ACD	>50	>50	36.66	<0.02	4.64
T257-31	AG	10	50	>50	6.5	
T250-4	AG	14	14.5	>50	14.5	12.14
T266-60	AG	<0.023	0.0304	0.02	0.079	1.92
236-8	AG	<0.023	0.103	0.32	0.037	5.4
271-11	AG	3.31	4.3	18.76	<0.02	14.67
242-14	AG	>50	>50	>50	0.12	>50
928-28	AG	>50	>50	>50	0.18	2.42
T33-7	AG	●	●	●	0.15	●
T278-50	AG	●	●	●	4.1	0.84
T25118	AG	●	●	●	0.71	0.63
R1166.c07	AE	<0.02	0.02	1.24	13.81	10.39
R3265.c06	AE	0.04	0.05	0.12	35.27	12.13
C2101.c01	AE	<0.02	<0.02	0.03	>50	<0.023
SS1196	B	10.98	29.64	7.05	0.71	<0.023

**Bal26**	B	0.743	2.65	0.36	0.1	0.213
**TRJO4551.58**	B	48.7	>50	>50	11.8	0.565
**AC10.0.29**	B	0.238	0.732	0.2	>50	•
**IIIb**	B	0.75	2	1.2	<1	0.84
**THRO4156.18**	B	0.94	2.275	1.6	0.85	1.31
**6535.3**	B	>50	>50	11.97	2.78	33.85
**QH0692.42**	B	0.3	0.49	0.11	0.13	•
**SC422661.8**	B	3.33	4.04	1.07	2.45	0.12
**PVO.4**	B	0.19	0.19	0.05	0.52	0.19
**TRO.11**	B	<0.02	<0.02	<0.02	3.39	0.63
**RHPA4259.7**	B	0.03	0.05	<0.02	0.07	0.1
**REJO4541.67**	B	0.24	0.38	0.19	0.05	0.97
**WITO4160.33**	B	8.63	14.36	0.07	1.17	1.01
**CAAN5342.A2**	B	1.57	2.55	0.88	10.85	1.19
**CH038**	BC	>50	>50	>50	<50	0.85
**CH181.12**	BC	4.6	5.7	1.9	0.86	7.99
**CH110.2**	BC	6	17.7	6.3	0.99	<0.023
**CH064.20**	BC	1.43	4.8	1.6	0.12	<0.1
**CH091.9**	BC	3.4	7.2	3	0.18	0.049
**CH119.10**	BC	•	•	•	1.1	•
**CH114.8**	BC	•	•	•	11.1	•
**CH115.12**	BC	•	•	•	>50	•
**CH120.6**	BC	•	•	•	>50	•
**CH070.1**	BC	•	•	•	>50	•
**CH111.8**	BC	•	•	•	1.4	0.534
**CH117.4**	BC	•	•	•	>50	•
**CNE19**	BC	0.08	0.12	0.04	0.37	0.101
**CNE20**	BC	>50	>50	>50	>50	0.13
**CNE21**	BC	0.19	0.28	0.07	2.44	•
**DU172**	C	0.293	0.891	0.51	31.4	0.193

**TV1.12**	C	>50	>50	>50	>50	0.21
**Du156.12**	C	0.368	1.597	0.55	0.43	•
**ZM135M.PL10a**	C	0.0876	0.27	0.11	4.35	0.284
**ZM109F.PB4**	C	0.0337	0.2	0.122	6.93	0.633
**96ZM651.02**	C	0.43	0.67	0.069	0.28	0.783
**93MW965.26**	C	0.19	0.48	0.21	2.09	0.801
**Du422.1**	C	0.36	0.47	0.4	0.64	0.958
**ZM197M.PB7**	C	41.86	>50	>50	6.17	1.51
**ZM214M.PL15**	C	<0.02	0.04	0.02	0.36	3.67
**ZM249M.PL1**	C	1.19	1.9	0.71	0.27	7.66
**6952.v1.c20**	CD	>50	>50	>50	1.02	14.35
**6811.v7.c18**	CD	0.14	0.27	0.12	0.28	23.77
**X2131_C1_B5**	G	14.38	18.85	3.96	0.56	0.12
**P1981_C5_3**	G	>50	>50	>50	>50	0.19
**X1632_S2_B10**	G	0.1	0.14	5.54	0.75	0.74
**X1254_C3**	G	>50	>50	>50	>50	0.97
**X2160_C25**	G	2.88	4.81	48.35	2.02	1.2
**X2088_C9**	G	>50	>50	>50	>50	0.126
**Viruses neutralized**		47	44	45	58	
**Viruses tested**		61	61	61	71	
**%**		77	72	74	81.7	
**Median IC50 μg/ml**		0.85	0.82	0.53	0.73	

Supplementary Table 3. IC50 values for B9 VHH and S54W mutant against nine viruses.
The S54W mutant was generated by site-directed mutagenesis as described in the materials and methods. VHH were titrated threefold from 50 µg/ml and incubated with the indicated pseudoviruses on TZM-bl assay. VHH IC50 titers were calculated using the XLFit4 software (IDBS). Highly potent neutralization (IC50 < 0.1µg/ml) is color-coded dark red, potent neutralization (0.1-1µg/ml) is color-coded red, intermediate neutralization (1-10µg/ml) is color-coded yellow, weak neutralization (10-50 µg/ml) is color-coded green.

Virus	Clade	B9	S54W	Fold increase
T266-60	AG	0.039	0.056	0.704
236-8	AG	0.056	0.079	0.710
RHPA4259.7	B	0.063	0.159	0.400
TRO.11	B	0.245	0.162	1.509
Du156.12	C	0.559	0.413	1.355
DU172	C	0.829	0.331	2.505
CH181.12	BC	1.532	0.811	1.889
CH110.2	AG	2.142	2.398	0.893
Bal26	B	2.993	0.200	14.995
SS1196	B	13.881	3.796	3.656
WITO4160c	B	30.301	7.487	4.047
TRJO4551.58	B	>50	37.289	
CH038	BC	>50	32.90	

Supplementary Table 4: Statistical analysis of sequence variation between immunized and naïve llamas.

The mean cluster size was found to be significantly larger for naive than immunized llamas. When considering only non-singleton clusters (i.e. clusters of sequences with 2 or more members) the average cluster size was also considerably larger for the naive llamas. The mean number of reads per unique sequence was also higher in the naive compared to the immunized samples highlighting the greater sequence diversity in the immunized samples. This was despite the total number of reads not varying significantly between either set of samples and further reinforced by the immunized animals generating significantly higher numbers of unique sequences per sample.

	Naïve llamas	Immunized llamas	p (Student's t-test)
Total mean cluster size	1.25*	1.05	<0.005
Mean cluster size for clusters of ≥2	5.3	2.7	<0.005
Mean number of reads per unique sequence	1.52	1.25	<0.005
Total numbers of unique sequences per sample*	1.8×10^5	1.5×10^5	<0.005
Total number of reads	2.3×10^5	2.1×10^5	0.6

*Normalized for total reads in each sample

Supplementary Figure 1: Post-immune sera anti-HIV activity.
(A) Threefold serial dilutions of llama sera were tested against the indicated pseudoviruses, starting at a 1:5 dilution in the 96-well plate the TZM-bl cell-based assay as described in the materials and methods. (B) Threefold serial dilutions of llama sera were tested against the indicated immunogens, starting at a 1:20 dilution. Binding was detected with HRP-conujugated anti-llama antibody. Serum samples were heat-inactivated to destroy complement by incubation at 56°C for 1 h before use.

(A)

	**92UG037**<br>A (2)	**Bal26**<br>B (1)	**96ZM0651.02**<br>C (2)	**R2**<br>B(nd)
**Llama 1**	1: 106	1: 20	1: 147	1:100
**Llama 3**	1: 25	1: 8	1: 100	1:27

(B)

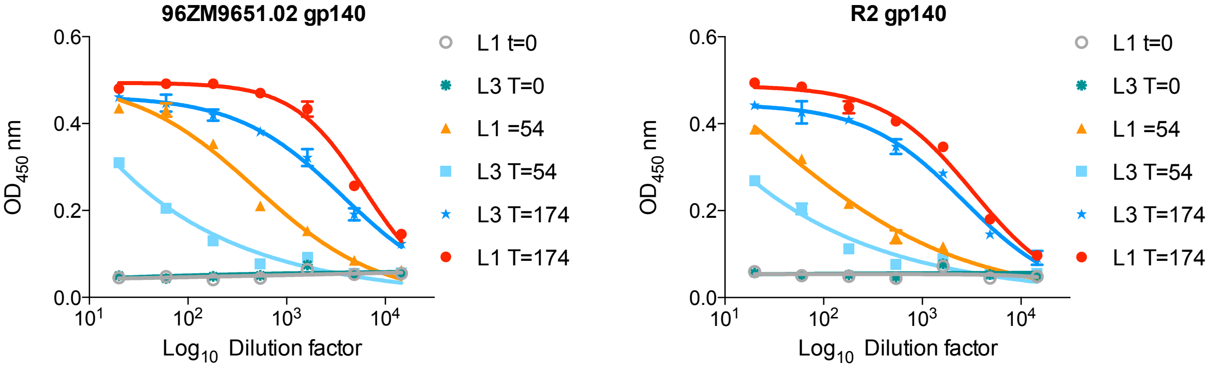

(C)

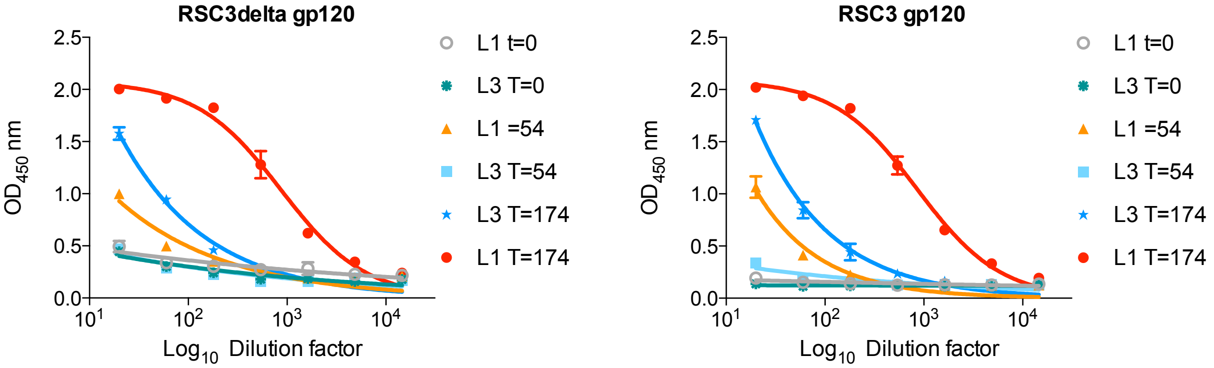

Supplementary Figure 2: VHH binding to HIV Env proteins.

VHH binding to **(A)** clade B gp140 BX08, **(B)** clade B gp41 IIIB and **(C)** clade A gp140 92UG037 was assessed by ELISA as described in the Materials and Methods. The positive control for gp140 binding was J3 [26] and that for gp41 binding 2H10 [32].

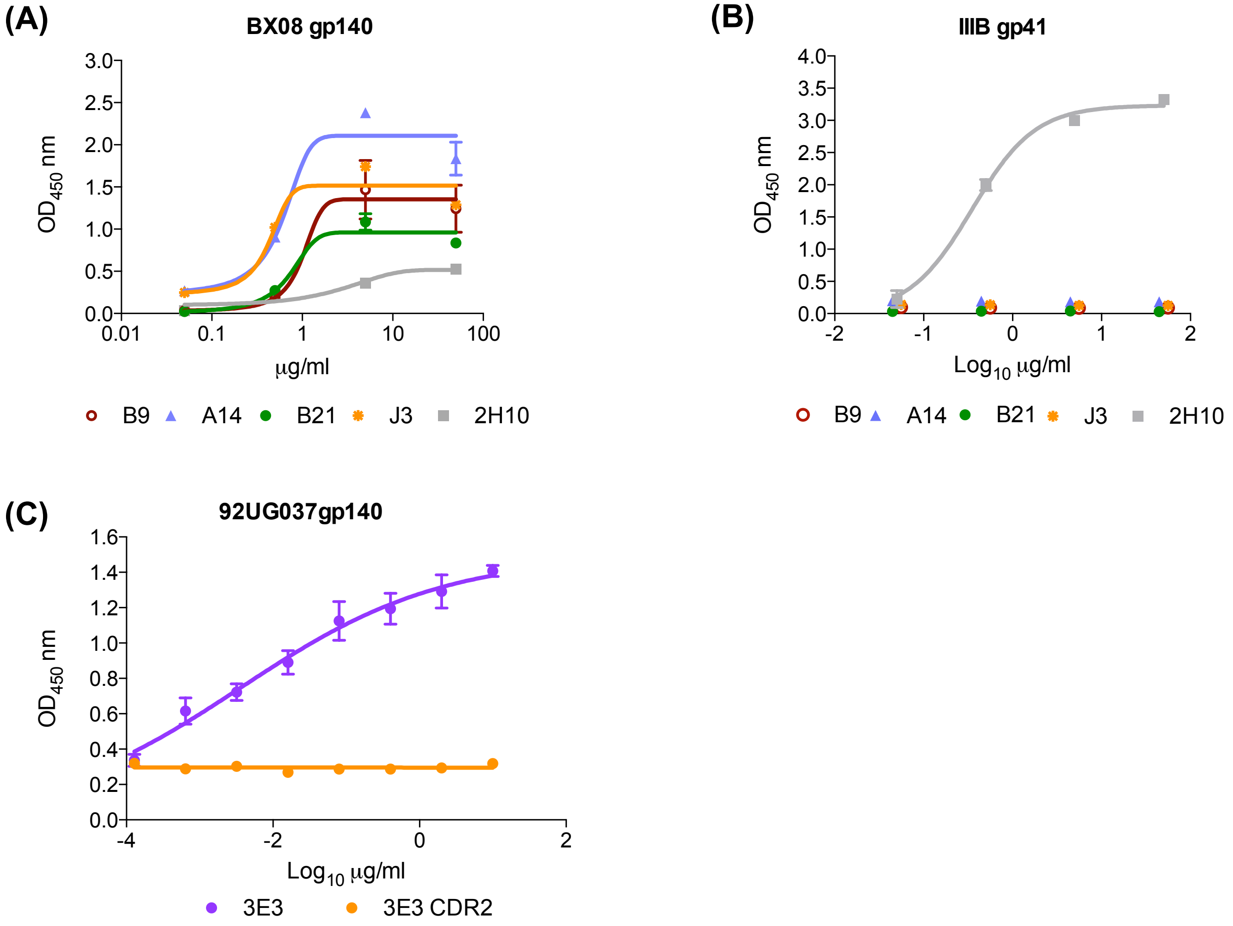

Supplementary Figure 3. VHH sequence alignments. Alignment of A14, B9, B21, 3E3, their germ line V genes and human V gene VH3-23*04 and VH1-2*02.

```
                        FR1-IMGT          CDR1-IMGT    FR2-IMGT       CDR2-IMGT
                          (1-26)            (27-38)    (39-55)          (56-65)

B9              EVQLVESGGGLVQAGGSLRLSCETSGFTFDDYGIGWFRQAPGKGREGISCISGS-GIAHY 59
A14             EVQLVESGGGLVQAGGSLRLSCAASGFSFNDYAIGWFRQTPGKEREGVSCLSSS-GMAHY 59
Vg              EVQLVESGGGLVQAGGSLRLSCAASGFTFDDYAIGWFRQAPGKEREGVSCISSSDGSTYY 60
3E3             EVQLVESGGGLVQPGGSLRLSCAASQFTLESYAIGWFRQAPGKDSEGVACISSS---TYY 57
Ve              QVQLVESGGGLVQPGGSLRLSCAASGFTLDYYAIGWFRQAPGKEREGVSCISSSDGSTYY 60
Vu              QVQLVESGGGLVQAGGSLRLSCAASGDTICISAMGWYRQAPGKERELVAAITSG-GSTNY 50
B21             EVQLVESGGGLVQAGGSLRLSCEASTSMFSIRAATWYRQAPGKQRELVANIDSE-GTTGY 59
VH3-23*04       EVQLVESGGGLVQPGGSLRLSCAASGFTFSSYAMSWVRQAPGKGLEWVSAISGSGGSTYY 60
VH1-2*02        QVQLVQSGAEVKKPGASVKVSCKASGYTFTGYYMHWVRQAPGQGLEWMGWINPNSGGTNY 60

                           FR3-IMGT
                           (66-104)

B9              GDSVKGRFTISSDNAKNTVYLQMNSLKPEDTGLYYCATTPFRCGNWRTTMGSWGQGTQVTVSS 122
A14             ADSVKGRFTIAYDNAKNTVYLQMNSLKPEDTAVYHCATHPFRCGNWRTVMGSWGQGTQVTVSS 122
Vg              ADSVKGRFTISSDNAKNTVYLQMNSLKPEDTAVYYCAA------------------------- 98
3E3             ADSVKGRFTISRDNAKNTVYLQMESLKPEDTAVYHCATSGAGSYCTLRAFGSWGQGTQVTVSS 120
Ve              ADSVKGRFTISRDNAKNTVYLQMNSLKPEDTAVYYCAT------------------------- 98
Vu              ADSVKGRFTISRDNAKNTVYLQMNSLKPEDTAVYYCNA------------------------- 98
B21             SDSVKGRFTISRDNTKKTVYLQMNSLKPEDTAVYSCNAVVTYN---MLVYDSWGQGTQVTVSS 119
VH3-23*04       ADSVKGRFTISRDNSKNTLYLQMNSLRAEDTAVYYCAK------------------------- 98
VH1-2*02        AQKFQGRVTMTRDTSISTAYMELSRLRSDDTAVYYCAR------------------------- 98
```

Supplementary Figure 4. Shared sequence identity with J3 relative to divergence from germ line for all naïve and immunized llamas.

Shared percentage identities with neutralizing VHH J3 and divergence from its inferred V gene Vt were calculated for all unique sequences from the seven control naïve llamas, and the four immunized llama including the J3-source llama 8. Each panel shows percentage identity for all sequences from the indicated llama plotted against divergence from Vt.

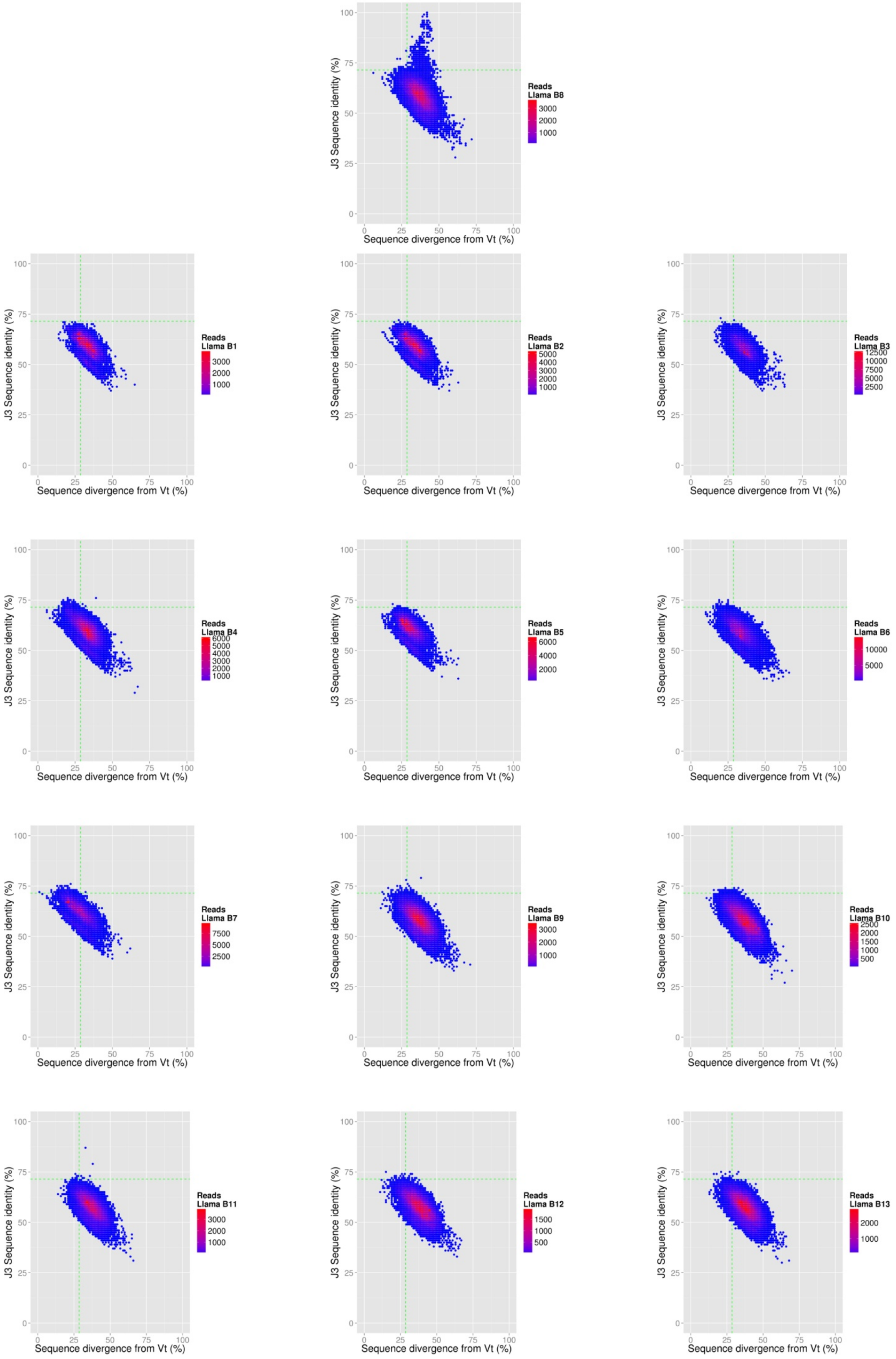

Figure S4. J3 identity of VHH repertoires of naïve llama and non-llama 8 immunized llamas.
